# Tolerogenic dendritic cells generated *in vitro* using a novel protocol mimicking mucosal tolerance mechanisms represent a potential therapeutic cell platform for induction of immune tolerance

**DOI:** 10.3389/fimmu.2023.1045183

**Published:** 2023-10-13

**Authors:** Gillian Dao Nyesiga, Lieneke Pool, Pavlos C. Englezou, Terese Hylander, Lars Ohlsson, Daniel Appelgren, Anette Sundstedt, Kristina Tillerkvist, Hanne R. Romedahl, Maria Wigren

**Affiliations:** ^1^ Idogen AB, Lund, Sweden; ^2^ Department of Biomedical Sciences, Faculty of Health and Society, Malmö University, Malmö, Sweden; ^3^ Department of Health, Medicine and Caring Sciences, Faculty of Medicine and Health Sciences, Linköping University, Linköping, Sweden

**Keywords:** tolerogenic dendritic cells (tolDCs), regulatory T cells (Tregs), regulatory B cells (Bregs), cell therapy, antigen-specific response, immune tolerance, antigen loading

## Abstract

Dendritic cells (DCs) are mediators between innate and adaptive immunity and vital in initiating and modulating antigen-specific immune responses. The most important site for induction of tolerance is the gut mucosa, where TGF-β, retinoic acid, and aryl hydrocarbon receptors collaborate in DCs to induce a tolerogenic phenotype. To mimic this, a novel combination of compounds – the synthetic aryl hydrocarbon receptor (AhR) agonist IGN-512 together with TGF-β and retinoic acid – was developed to create a platform technology for induction of tolerogenic DCs intended for treatment of several conditions caused by unwanted immune activation. These *in vitro*-generated cells, designated ItolDCs, are phenotypically characterized by their low expression of co-stimulatory and activating molecules along with high expression of tolerance-associated markers such as ILT3, CD103, and LAP, and a weak pro-inflammatory cytokine profile. When co-cultured with T cells and/or B cells, ItolDC-cultures contain higher frequencies of CD25+Foxp3+ regulatory T cells (Tregs), CD49b+LAG3+ ‘type 1 regulatory (Tr1) T cells, and IL-10-producing B cells and are less T cell stimulatory compared to cultures with matured DCs. Factor VIII (FVIII) and tetanus toxoid (TT) were used as model antigens to study ItolDC antigen-loading. ItolDCs can take up FVIII, process, and present FVIII peptides on HLA-DR. By loading both ItolDCs and mDCs with TT, antigen-specific T cell proliferation was observed. Cryo-preserved ItolDCs showed a stable tolerogenic phenotype that was maintained after stimulation with LPS, CD40L, or a pro-inflammatory cocktail. Moreover, exposure to other immune cells did not negatively impact ItolDCs’ expression of tolerogenic markers. In summary, a novel protocol was developed supporting the generation of a stable population of human DCs *in vitro* that exhibited a tolerogenic phenotype with an ability to increase proportions of induced regulatory T and B cells in mixed cultures. This protocol has the potential to constitute the base of a tolDC platform for inducing antigen-specific tolerance in disorders caused by undesired antigen-specific immune cell activation.

## 1 Introduction

Dendritic cells (DCs) play a crucial role in the initiation of immune responses to harmful pathogens and tumors but are also key players in the process of tolerance induction in the periphery by various mechanisms. Residing in tissues and organs, DCs continuously take up and process antigens, both foreign and self-antigens, from their microenvironment. Peptide fragments of such processed antigens are presented to T cells after DCs migrate into the draining lymph nodes (1). The level of DC maturation is an important factor determining the direction of an immune response, stimulation vs tolerance, and whether naïve T cells will be activated or deleted, or if effector T cells or regulatory T cells (Tregs) will be induced or activated (2). Tolerogenic DCs (tolDCs) typically express low levels of markers associated with maturation and activation of DCs, such as the co-stimulatory molecules CD80 and CD86, and the activation markers CD83 and human leukocyte antigen (HLA)-DR. Moreover, tolDCs can express high levels of tolerance-associated molecules such as immunoglobulin-like transcript (ILT)3, CD103, latency-associated peptide (LAP), indoleamine 2,3-dioxygenase (IDO), HLA-G, programmed death-ligand (PD-L)1, and PD-L2 (3). DCs co-expressing CD141 and glycoprotein A repetition predominant (GARP) have tolerogenic functions and enhanced capacity to induce Tregs (4).

TolDCs are continuously induced *in vivo* to maintain peripheral tolerance (5). The major site for induction of tolerance is the mucosa along the gastrointestinal tract. However, DCs with a tolerogenic phenotype and function can also be generated *in vitro* by several different means. They are typically generated from monocytes or other precursor cells by introducing one or more tolerance-inducing compounds at defined time points during their differentiation into DCs (3, 6). For example, treatment with dexamethasone (Dex) and/or the activated form of vitamin D3 (1,25-dihydroxyvitamin D3; 1α,25(OH)_2_D_3_; VitD3) has frequently been used to induce human tolDCs (7–9). The use of interleukin (IL)-10, rapamycin, Dex together with vitamin A, and inactivation of NF-kB with Bay11-7082 are further examples of how human tolDCs can be induced *in vitro* (10–14).

CD103-expressing DCs constitute a non-lymphoid subset that is vital in generating immunity and maintaining homeostasis in the gut, lungs, and other mucosal sites. These DCs play a crucial role in gut homeostasis via induction of Tregs in the mesenteric lymph nodes (MLNs) in the presence of all-trans retinoic acid (RA) and transforming growth factor beta (TGF-β) (15–17). TGF-β is a pleiotropic cytokine important in establishing and maintaining immunological tolerance by regulating the activation and differentiation of T cells. Biologically active TGF-β is associated with its pro-peptide LAP from which it needs to be released to bind to its associated receptor. The expression of extracellular LAP on DCs has been shown to control the fate of naïve T helper cells (18, 19). An *in vitro* study showed that human DCs treated with RA induced functional and suppressive IL-10-producing Tregs (20). This study also showed that RA-treated DCs in the presence of TGF-β induced a shift towards more forkhead box P3 (Foxp3)-expressing Tregs, suggesting that RA and TGF-β act synergistically in the induction of tolerance in mucosal sites.

Activation of the aryl hydrocarbon receptor (AhR) is yet another way to obtain DCs with tolerogenic properties, including the induction of Tregs (21–23). The AhR can be activated or inactivated by endogenous and exogenous ligands. Different types of AhR ligands may exert distinct effects after interaction with their receptor protein (24, 25). AhR is widely expressed by different immune cells in the gut where it has a protective role (26). AhR activation was shown to inhibit DC maturation, assessed as reduced production of tumor necrosis factor alpha (TNF-α), IL-1β, IL-6, and IL-23; increased secretion of IL-10; and decreased expression of HLA-DR, CD83, and CD86 (24, 27). Furthermore, AhR activation in the gut has been shown to increase regulatory mediators, e.g. IL-10, IL-22, prostaglandin E2 (PGE2), and Foxp3, together with production of metabolites from commensal bacteria which may also activate AhR (26).

Induction and activation of regulatory immune cells using tolDC therapies is an expanding technology, which has been clinically tested in autoimmune diseases, anti-drug antibody formation, and organ rejection in transplantation, all of which are caused by unwanted immune activation (5, 28–31). TolDCs have in some, but not in all instances, been loaded with disease-specific antigen(s) to enhance their ability to directly interact with and inactivate self-reactive T cells that cause tissue damage (28). It has been described for *in vivo* investigations that tolDCs loaded with a disease-specific antigen more effectively inhibit disease progression compared to tolDCs not loaded with an antigen (32). In addition, an antigen-specific tolDC therapy, compared to non-specific tolerance induction, could potentially reduce the risk of unwanted immune tolerance to non-disease antigens, e.g. pathogenic viruses and cancer cells.

Inspired by the aforementioned pathways of tolerance induction in the gastro-intestinal tract, we tested whether the synthetic AhR agonist IGN-512, RA, and TGF-β could be used, either separately or in combinations with each other, to *in vitro* generate tolDCs like the potent naturally-occurring tolerogenic cells present in the mucosa and gut-draining lymph nodes. We found that a combination of all three could generate a unique tolDC phenotype that from here on will be referred to as ItolDCs. Antigen-specific T cell proliferation was investigated, as was the capacity to resist maturation in an inflammatory environment and in cell-to-cell contact with other lymphocyte populations and compared to other *in vitro* generated DC subtypes. ItolDCs showed stability, function, and capacity to induce immune regulatory cells that was similar or even surpassed that of tolDC induced *in vitro* using Dex and VitD3. To our knowledge, no human tolDCs intended for clinical use have been generated using a method analogous to the natural tolerance-inducing mechanisms at mucosal sites. Our long-term goal is now to develop these cells into a tolerogenic dendritic cell platform that can be used clinically to induce tolerance in an antigen-specific manner for the treatment of autoimmune diseases, organ rejection, and drug resistance caused by anti-drug antibodies.

## 2 Materials and methods

### 2.1 Cell isolation and processing

Leukocyte concentrates of healthy donors were purchased from the local blood bank at Skåne university hospital, department of Clinical Immunology and Transfusion Medicine, Lund, Sweden with the approval of the Swedish Ethical Review Authority (Etikprövningsmyndigheten). Peripheral blood mononuclear cells (PBMCs) were isolated by density gradient centrifugation using Lymphoprep™ and SepMate™-50 (STEMCELL Technologies, Vancouver, Canada). CD14+ monocytes were magnetically enriched from the isolated PBMCs using anti-CD14 microbeads (Miltenyi Biotec, Bergisch Gladbach, Germany). CD4+ T cells, B cells or total T cells (CD4+ and CD8+) were isolated from PBMCs via immunomagnetic negative selection using EasySep™ Cell Isolation Kits (STEMCELL Technologies). All cells were isolated according to manufacturer’s instructions. Isolated PBMCs, PBMCs depleted of CD14+ cells, and generated DCs were separately cryopreserved in CryoStor^®^ CS10 (STEMCELL Technologies).

### 2.2 Generation of DCs

Isolated CD14+ monocytes were cultured for 7 days in Costar™ cell culture plates (Corning™ Incorporated, New York, NY, USA) at 1.56×10^6^ cells/ml in complete GMP DC medium (CellGenix^®^ GMP Dendritic Cell Medium, Sartorius CellGenix GmbH, Freiburg, Germany) supplemented with 10 mM HEPES, 1x GlutaMAX™, 5 U/ml Penicillin, 5 µg/ml Streptomycin, 50 ng/ml recombinant human granulocyte-macrophage colony-stimulating factor (GM-CSF), and 50 ng/ml IL-4 (both from PeproTech, Cranbury, NJ, USA). All cultures were supplemented on Day 3 with complete GMP DC medium with both 50 ng/ml GM-CSF and 50 ng/ml IL-4 and on Day 6 with complete GMP DC medium alone. For immature DCs (imDCs), no further substances were added to the culture. For mature DCs (mDCs), the cells were matured 22 h prior to harvest with 0.5 μg/ml Lipopolysaccharides (LPS) from *Escherichia coli* O128:B12 (Merck KGaA, Darmstadt, Germany) or with a maturation cocktail consisting of 10 ng/ml TNF-α, 10 ng/ml IL-1β, 1000 U/ml IL-6 (all from PeproTech), and 1 μg/ml PGE2 (Merck KGaA) (designated TIP6 cocktail). For Dex/VitD3-tolDC, the cultures were supplemented with 100 nM VitD3 (STEMCELL Technologies) and 100 nM Dex (Merck KGaA) on Day 3. For generation of ItolDCs, CD14+ cells were cultured with 10 nM of the synthetic AhR agonist IGN-512 (Idogen AB, Lund, Sweden) from the start. The cultures were further supplemented with 10 nM IGN-512 and 10 ng/ml TGF-β (PeproTech) on Day 3, and with 2 μM all-trans RA (Merck KGaA) on Day 6. For generation of LPS-stimulated ItolDCs, LPS was added 2 h after the addition of RA. For the TT-specific T cell proliferation assay, ItolDCs were generated from monocytes isolated from cryopreserved PBMCs. ItolDCs were loaded with antigen 2 h after the addition of RA, either with 15 or 30 nM Factor VIII (FVIII) (Kovaltry; Bayer, San Francisco, CA, USA) or with 30 nM recombinant TT (Merck KGaA). On Day 7, the phenotype of DCs was assessed using flow cytometry. To prevent carry-over of the immunomodulatory molecules, all DC cultures were washed 3 times with PBS after harvest before further cultivation and analysis. All cell cultures were performed at 37°C with 5% CO_2_ in the atmosphere. The information of ItolDCs provided here adhere to the reporting framework MITAP (minimum information model for tolAPC) (33).

### 2.3 Biological activity of IGN-512 using RT-qPCR

Upregulation of AhR activation-associated genes by IGN-512 was analyzed using reverse transcription (RT) quantitative real-time PCR (qPCR) and was compared to other known AhR agonists TCDD (2,3,7,8-Tetrachlorodibenzo-p-dioxin) (Supelco, Bellefonte, PA, USA) and FICZ (5,11-Dihydroindolo[3,2-b]carbazole-6-carboxaldehyde) (Tocris Bioscience, Bristol, United Kingdom). Either TCDD or FICZ was added at 10 nM to the cultures in place of IGN-512 during the DC generation. During the generation of ItolDCs on Day 2 and at the end of generation on Day 7, the cells were harvested, counted, and washed twice with PBS. The pelleted cells were resuspended in Buffer RLT Plus for RNA isolation using RNeasy Plus Mini kit (Qiagen, Hilden, Germany) and subsequent RT-qPCR analysis. Cells from Day 2 were stored at -80°C prior to RNA isolation. RT was performed on 1 µg of RNA, and qPCR analysis was run in 5 µl reactions in duplicates to analyze the following genes: *UGT1A1*, *CYP1A1*, *CYP1A2*, *CYP1B1*, *CYP2B6*, *NQO1*, *TIPARP*, and *AHRR*. *B2M* and *RPL13a* were used as housekeeping genes. RT-qPCR was performed using BioRad CFX384 RealTime System, C1000 Thermal Cycler and CFX Maestro software (all from Bio-Rad Laboratories, Hercules, CA, USA). Results were calculated as 2^-ΔCT^ values (normalized to the housekeeping gene expression).

### 2.4 Cytokine profile of ItolDC

Supernatants from the DC cultures were collected on Day 7 and cytokine analysis was performed according to manufacturer’s protocol using a magnetic bead-based multiplex assays (Bio-Techne, Minneapolis, MN, USA) with the following analytes: IL-1β, OPN, IL-6, IL-10, IL-12p70, IL-16, BDCA-3, CD163, IL-36, TRAIL, PD-L1, and TNF-α. Samples were analyzed using a Luminex^®^ MAGPIX^®^ Analyzer (Luminex Corporation, Austin, TX, USA).

### 2.5 Mixed lymphocyte reaction

For mixed lymphocyte reactions (MLRs), DCs (10^4^/well) were co-cultured with allogeneic CD4+ T cells at a ratio of 1:10 (DC:CD4+ T cells) or CD14-depleted PBMCs at a ratio of 1:5 (DC:PBMC depleted of CD14+ cells) in complete RPMI (RPMI 1640 medium supplemented with 10 mM HEPES, 1x GlutaMAX™, 5 U/ml Penicillin, 5 μg/ml Streptomycin, and 10% heat-inactivated dialyzed fetal bovine serum (FBS) in 96-well plates (Thermo Fisher Scientific). Cell proliferation was determined by tritiated ([^3^H]) thymidine incorporation, carboxyfluorescein diacetate succinimidyl ester (CFDA) dilution, or intranuclear Ki-67. For thymidine incorporation, MLRs were on Day 5 further cultured with 1 µCi [^3^H]-thymidine (Perkin Elmer, Waltham, MA, USA) for 18 h. For CFDA-dilution, isolated CD4+ T cells were labelled with 0.3 µM CFDA (Thermo Fisher Scientific) and proliferation was assessed after 7 days of MLR using flow cytometry. For detection of CD49b+LAG3+ Tr1 cells, MLRs were cultured for 7 days, and for detection of CD25+Foxp3+ Tregs, cells were incubated with 20 U/ml IL-2 (PeproTech) for 7 additional days. For assessment of the expression of intracellular cytokines of DC-primed CD4+ T cells, the cultures were stimulated for 3 h with a pre-mixed Cell Activation Cocktail (BioLegend) consisting of 40.5 µM phorbol-12-myristate 13-acetate (PMA) and 669.3 µM ionomycin followed by 2 h stimulation with 2 µM monensin (BioLegend) on Day 7 of MLR prior to extracellular and intracellular flow cytometric staining.

### 2.6 Autologous B cell cultures with DCs and T cells

To induce Bregs, DCs (10^4^/well) were cultured in flat-bottomed 96-well plates (Eppendorf, Hamburg, Germany) in complete RPMI overnight at 37°C, and isolated autologous B cells were added to the cultured DCs the next day with or without autologous T cells at a ratio of 1:4:4 (DC:B cell:T cell) in fresh medium with 0.25 µM CpG-ODN (InvivoGen, Toulouse, France). After 7 days of co-culture, proliferation of T cells was assessed by the expression of intranuclear Ki-67. To identify IL-10-producing CD19+ Bregs, co-cultures were incubated for 48 h and thereafter stimulated for 3 h with Cell Activation Cocktail followed by 1 h stimulation with 2 µM monensin prior to intracellular IL-10 staining. The cells were assessed using flow cytometry.

### 2.7 Antigen-specific autologous assays

For assessment of antigen-specific T cell proliferation, mDCs were matured using a mix of 100 ng/ml PGE2, 2.5 ng/ml IL-1β, 100 IU/ml IL-6, 300 IU/ml IFN-α (Miltenyi Biotec), 100 IU/ml IFN-γ, 5 ng/ml TNF-α, 2 ng/ml Polyinosinic:polycytidylic acid (Poly:IC) (Merck KGaA), and 100 ng/ml CD40L (PSI Inc., Glendale, CA, USA) (designated NIH low cocktail) 2 h after antigen loading. DCs (10^4^/well) were plated with CD4+ T cells at a 1:10 ratio in CTS™ OpTmizer™ T Cell Expansion medium (Thermo Fisher Scientific) for 7 days. T cell proliferation was assessed using Ki-67 expression in CD4+ T cells. Donors were considered responsive to TT when the mean value of six replicates were >2 times higher than the background proliferation induced by that of mDC without antigen loading. One donor out of six was non-responsive to TT and excluded.

### 2.8 Antigen-presentation via HLA-DR

To identify HLA-DR-bound FVIII peptides after antigen uptake by ItolDCs, eluted peptides from ItolDCs were analyzed using mass spectrometry as previously described (34). Briefly, cryopreserved ItolDCs with and without FVIII loading were thawed, lyzed, and supernatant was incubated overnight at 4°C with antibody L243-coupled CNBr Sepharose resin (anti-HLA-DR) (Amersham Biosciences, Buckinghamshire, UK). Eluted peptides were analyzed by mass spectrometry using Orbitrap Fusion Tribrid mass spectrometer (Thermo Fisher Scientific Inc., Bremen, Germany). Peptide were identified using Proteome Discoverer 1.4 (Thermo Scientific, Bremen, Germany). Raw Xcalibur files were screened against the UniprotKB nonredundant protein database 25.H_sapiens.fasta (53,784 non-redundant entries actually searched), using Proteome Discoverer release version 1.1 software (Thermo Scientific). A mass deviation of 20 ppm, a fragment mass tolerance of 0.8 Da, and a false positive discovery rate of 95% was allowed. All identified FVIII-derived peptides with high and medium confidence were grouped and aligned using Clustal Omega (EMBL-EBI) for each donor.

### 2.9 Assessment of maturation resistance

ItolDCs were re-seeded at 1×10^6^ cells/ml in complete GMP DC medium either directly after harvest or after freeze-thawing and restimulated for 24 h with low or physiologically relevant concentrations of LPS (1 ng/ml – 0.5 µg/ml), CD40 ligand (CD40L) (10 pg/ml – 1 ng/ml) (PeproTech), or a pro-inflammatory cocktail consisting of 1 ng/ml of each cytokine: TNF-α, IL-1β, IFN-α, IL-8 (Peprotech), and IL-6 (Peprotech). DCs were harvested on Day 7 and cultured with allogeneic CFDA-labeled PBMCs at a 1:7.5 (DC:PBMC) ratio in complete RPMI. The phenotype of the DCs was analyzed before they were added to the co-culture with PBMCs, and directly after, and at 1, 2, and 4 h after start of the co-culture.

### 2.10 Antibodies and flow cytometry analyses

Cell viability was assessed using LIVE/DEAD™ Fixable Green or Near-IR Dead Cell Stain Kits (Thermo Fisher Scientific). To assess the phenotype of DCs, the following antibodies were used: CD11c Brilliant Violet (BV) 421 (REA618), CD83 Vio^®^ Bright Fluorescein isothiocyanate (FITC) (REA714), GARP Vio^®^ Bright B515 (REA166), CD141 Phycoerythrin (PE) (REA674), CD80 PE (REA661), ILT3 Allophycocyanin (APC) (REA141), GARP PE (REA166), CD11c APC Cyanine (Cy) 7 (REA618) (Miltenyi Biotec), LAP APC (TW4-2F8), CD83 APCCy7 (HB15e), HLA-DR PECy5 (L243), HLA-DR BV510 (L243), ILT3 PE (ZM4.1), PD-L1 PECy7 (29E.2A3), LAP APC (TW4-2F8) (BioLegend, San Diego, CA, USA), ILT3 APC (ZM4.1), CD103 PECy7 (B-Ly7), CD86 FITC (MAC08601), CD86 PECy7 (IT2.2), CD141 BV510 (1A4), CD103 PECy7 (B-Ly7), IDO eFlour 660 (eyedio) (Thermo Fisher Scientific), CD11c APC (B-Ly6), CD11c FITC (B-Ly6), GARP BV421 (7B11), HLA-DR PECy7 (G46-6), CD83 BV421 (HB15-e), and CD80 PECy7 (L307.1) (BD Biosciences, Franklin Lakes, NJ, USA). Different antibodies and instrument settings were used in the gathered data; therefore, MFI was recalculated to percentage relative to Dex/VitD3-tolDC. For T cell proliferation and identification of Tr1 cells, the following antibodies were used: CD25 BV421 (M-A251), CD4 BV510 (A161A1), CD49b PE (P1E6-C5), LAG3 PECy7 (11C3C65) (BioLegend), and Ki-67 APC (B56) (BD Biosciences). For identification of Tregs, Ki-67 is replaced with Foxp3 Alexa Flour (AF) 647 (259D/C7) (BD Biosciences). To identify regulatory B cells (Bregs), the following antibodies were used: IL-10 BV421 (JES3-9D7) (BioLegend), CD11c PE (BU15), CD19 PECy7 (HIB19), and CD4 APC (RPA-TA) (all from Thermo Fisher Scientific). Proliferation of T cells cultured with B cells was assessed using the following antibodies: CD4 BV421 (A161A1) (BioLegend), CD8 Brilliant™ Blue (BB) 515 (RPA-T8), CD3 Peridinin-Chlorophyll-Protein (PerCP) Cy5.5 (UCHT1), Ki-67 APC, and CD19 PE-Cy7 (HIB19) (Thermo Fisher Scientific). The following antibodies were used for intracellular cytokine staining: IL-2 PECy7 (MQ1-17H12), IL-10 BV421 (JES3-9D7), IL-17A PE (BL168), interferon (IFN)-ɣ APC (4S.B3) (BioLegend), CD45 BV510 (HI30), and CD4 APCCy7 (RPA-T4) (BD Biosciences). For intracellular staining, cells were fixed and permeabilized using eBioscience™ Foxp3/Transcription Factor Staining Buffer Set (Thermo Fisher Scientific) and labelled with adequate amounts of antibodies. For the assessment on Day 7 of uptake of FVIII, ItolDCs were labelled intracellularly using anti-FVIII conjugated with AF488 (Sanquin, Amsterdam, Netherlands). For the assessment of uptake of tetanus toxoid (TT), ItolDCs were loaded with recombinant TT that was conjugated with AF488 by Innovagen (Lund, Sweden), and 100 nM AF488-labeled TT was loaded on Day 3 and assessed on Day 7. The samples were stored at 4°C and acquired within 2 weeks using the flow cytometer MACSQuant Analyzer 10 (Miltenyi Biotec) and FlowJo version 10 (BD Biosciences).

### 2.11 Statistical analyses

Statistical analysis was performed using Prism version 9 (GraphPad Software, San Diego, CA, USA). Data are presented as mean, mean with standard deviation (SD), or mean with standard error of the mean (SEM). T-test was used for comparisons of the mean of two groups, and one-way ANOVA with *post-hoc* testing was used for comparison of multiple groups. Statistical significances are presented as ns=non-significant, *=p<0.05, **=p<0.01, ***=p<0.001, and ****=p<0.0001.

## 3 Results

### 3.1 Characterization of the tolerogenic phenotype of ItolDCs

The ItolDCs were generated using a mechanism analogous to the natural tolerance-inducing mechanisms described in the gut, employing the synthetic AhR agonist IGN-512, the pleiotropic cytokine TGF-β and the vitamin A metabolite RA. In the development of a novel protocol for the generation of ItolDCs, the following six criteria were assessed and fulfilled in comparison to mDCs: (1) decreased expression on the DCs of the activation markers HLA-DR, CD83, and CD86, (2) increased expression of tolerance-associated markers, e.g. ILT3 and CD103, (3) reduced production of pro-inflammatory cytokines, (4) decreased ability to stimulate T cell proliferation, (5) resistance to inflammatory stimulation, and finally, (6) potential to increase the proportions of regulatory lymphocytes. Throughout the studies, the tolDCs were compared to control DCs generated from monocytes from the same donor. The control DCs used for these comparisons were imDCs, mDCs, or tolDCs induced with Dex and VitD3 (Dex/VitD3-tolDCs).

**Figure 1:**
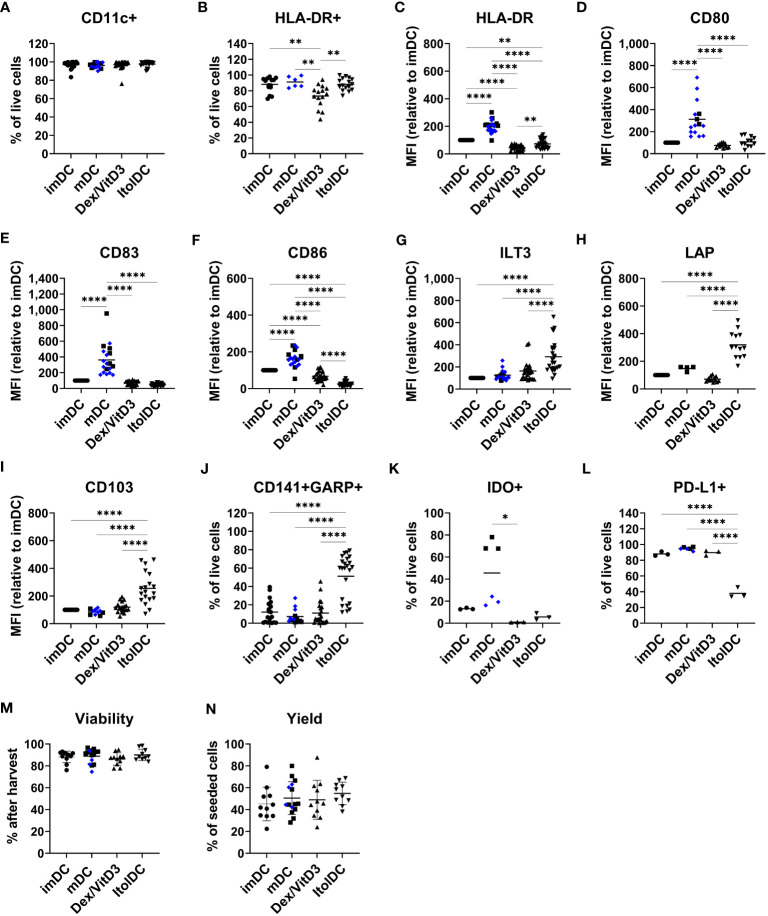
ItolDC phenotype assessed using flow cytometry in comparison to imDCs, mDCs matured with either LPS (blue diamond, ♦) or TIP6 cocktail (black square, ■) and Dex/VitD3-tolDCs. The data set shows **(A)** frequency of CD11c and **(B)** HLA-DR, **(C-I)** MFI of HLA-DR, CD80, CD83, CD86, ILT3, LAP, and CD103 **(J)** frequencies of GARP and CD141 double-positive cells, **(K)** IDO+ cells, **(L)** PD-L1+ cells, and **(M, N)** viability and yield at the end of DC differentiation. Frequencies and MFIs were determined on live cells. MFI values were calculated as relative to imDC, where MFI levels of imDC for each donor was set to 100. Data are presented as individual values with mean (A with n=19-27, B with n=6-16, C with n=25-28, D with n=12-15, E-G with n=19-25, H with n=4-13, I with n=10-19, J with n=13-23, K-L with n=3-6, and M-N with n=10-15, and one-way ANOVA with *post-hoc* testing was used for statistical comparisons. Statistical significances are presented as *=p<0.05, **=p<0.01, and ****=p<0.0001.

The phenotype of ItolDCs was compared to the phenotypes of imDCs, mDCs (matured with either LPS or TIP6 cocktail), and Dex/VitD3-tolDCs. The frequencies of different markers on DCs were gated on live cells (**Supplementary Figure 1**) and the geometric mean fluorescence intensity (MFI) levels, i.e. expression levels, were assessed on live cells. Similar high frequencies of CD11c and HLA-DR were observed on all DC types (**Figures 1A, B**). In addition, the expression levels of HLA-DR on ItolDCs were markedly lower than on mDCs (**Figure 1C**). Importantly, ItolDCs expressed lower levels of CD80, CD83, and CD86 compared to mDCs, assessed both as frequencies and as MFIs (**Figures 1D–F** and **Supplementary Figures 2A–C**).

The two molecules ILT3 and LAP, which are associated with tolerance induction, and CD103 were expressed by all four DCs subtypes (**Supplementary Figures 2D–F**). However, the expression levels of ILT3, LAP, and CD103 were significantly higher on ItolDCs compared to all control DCs (**Figures 1G–I**). The frequency of ILT3, LAP, and CD103 were higher for ItolDCs compared to the controls except for ILT3 where Dex/VitD3 were comparable to ItolDCs (**Supplementary Figures 2D–F**). Moreover, co-expression of CD141 and GARP were more frequent in ItolDCs compared to the three control DCs (**Figure 1J**). Both the frequency and MFI levels of GARP were also higher than the controls (**Supplementary Figures 2G, H**). ItolDCs had higher frequencies of CD141 compared to mDCs but not higher MFI levels (**Supplementary Figures 2I, J**). Both the frequencies and the expression levels of the tolerance-associated molecules IDO and PD-L1 were low for ItolDCs compared to mDCs matured with the TIP6 cocktail (**Figures 1K, L** and **Supplementary Figures 2K, L**). ItolDCs have similar or lower expression levels of CD80, CD83, and CD86 (**Figures 1D–F**) compared to Dex/VitD3-tolDCs but higher expression levels of ILT3, CD103, and LAP (**Figures 1G–I**). The viability of ItolDCs was on average 90% with a yield of approximately 55% (**Figures 1M, N**).

To get more insight into the effect of IGN-512, TGF-β, and RA, the kinetics of different molecules was studied during the differentiation from monocytes to ItolDCs (**Supplementary Figure 3**). The data showed that the frequency of CD11c+ cells was high for all donors during the whole differentiation period (**Supplementary Figure 3A**). However, the MFI level of CD11c increased over time during the differentiation and reached the highest level at the end of the culture (**Supplementary Figure 3B**). The frequency of HLA-DR+ cells increased over time, whereas cells lost CD14 expression within 3 days (**Supplementary Figures 3C, D**). The frequency of CD86+ cells was close to 100% at the start of the culture but dropped during the differentiation phase to its lowest frequency on Day 6 and partly recovered by Day 7 (**Supplementary Figure 3E**). Monocytes showed no expression of CD83, but the differentiating cells gained the highest frequency of CD83+ cells by Day 3, which was thereafter slightly decreased until Day 6 (**Supplementary Figure 3F**). The frequency of ILT3+ monocytes varied a lot between donors but reached the highest by Day 7 (**Supplementary Figure 3G**). Monocytes showed no or low frequencies of cells expressing LAP, CD103, GARP, and CD141, but the frequency increased during the differentiation period (**Supplementary Figures 3H–K**). Expression of LAP and co-expression of CD141 and GARP were induced from Day 6 (**Supplementary Figures 3H, L**), i.e. after addition of RA. The kinetics of the generation of ItolDCs show how the addition of the different immunomodulatory factors at different timepoints contributes to the phenotype of ItolDCs during the 7-day differentiation period (**Supplementary Figure 3**).

Each compound contributes to the tolerogenic phenotype and function of ItolDCs (**Supplementary Figures 3**–**6**). The separate compounds had no impact on HLA-DR expression, but in comparison to imDCs, generating DCs using only IGN-512 reduced the frequency of cells expressing the co-stimulatory molecule CD86, and generating DCs using TGF-β alone decreased the frequency of cells expressing the activation marker CD83 while generating DCs using only RA increased the MFI levels of the tolerance-associated marker ILT3 (**Supplementary Figures 4A** and **5A**). However, these changes towards a tolerogenic phenotype were not stable when stimulated with LPS (**Supplementary Figures 4A** and **5A**). Although DCs generated using IGN-512 paired with RA showed an increase in frequency of cells expressing the tolerance-associated marker CD103 and a decrease in frequency of CD83+ cells compared to imDCs, these cells were not resistant to LPS stimulation as the frequency of cells expressing CD83 and CD86, respectively, increased when stimulated with LPS (**Supplementary Figures 4B** and **5B**). In comparison to imDCs, DCs generated using IGN-512 together with TGF-β, reduced the frequency of cells expressing both CD83 and CD86, and although the frequency of cells expressing these markers increased after LPS stimulation, the frequencies were still significantly lower compared to the LPS-stimulated DC control (**Supplementary Figures 4C** and **5C**). This indicates that when combining IGN-512 and TGF-β, the generated DCs acquired resistance to inflammatory stimulation. LPS-stimulation of DCs generated using IGN-512 alone appeared to have increased IL-23 secretion (13.7 ng/ml) but was not significantly different from the IL-23 secretion in LPS-stimulated DCs generated using IGN-512 combined either with RA (6.4 ng/ml) or with RA and TGF-β (8.4 ng/ml) (**Supplementary Figure 6A**). When DCs generated using different combinations of IGN-512, TGF-β, and RA were cultured with T cells, it was shown that DCs generated using a combination of all three compounds, i.e. ItolDCs, induced lower T cell proliferation compared to DCs generated without IGN-512, i.e. with RA and TGF-β (**Supplementary Figure 6B**), and the highest frequencies of CD25+Foxp3+ Tregs (**Supplementary Figure 6C**). Additionally, DCs generated with the combination of the three compounds induce on average the lowest T cell proliferation (**Supplementary Figure 6B**). Overall, the combination of all three compounds generated a tolDC with a more tolerogenic phenotype and function compared to when the compounds were used separately or in different combinations of two.

Together, these data showed that ItolDCs have lower expression levels of markers associated with DC activation compared to mDCs and higher expression levels of the tolerance-associated molecules ILT3, CD103, and LAP compared to imDCs and mDCs. ItolDCs have a phenotype like Dex/VitD3-tolDCs except that ItolDCs have higher MFIs of the assessed markers associated with tolerance. Further, the kinetics data showed that the tolerogenic phenotypic profile of the cells evolved during the 7-day-differentiation period.

### 3.2 AhR activation-associated gene expression induced by the AhR agonist IGN-512

**Figure 2:**
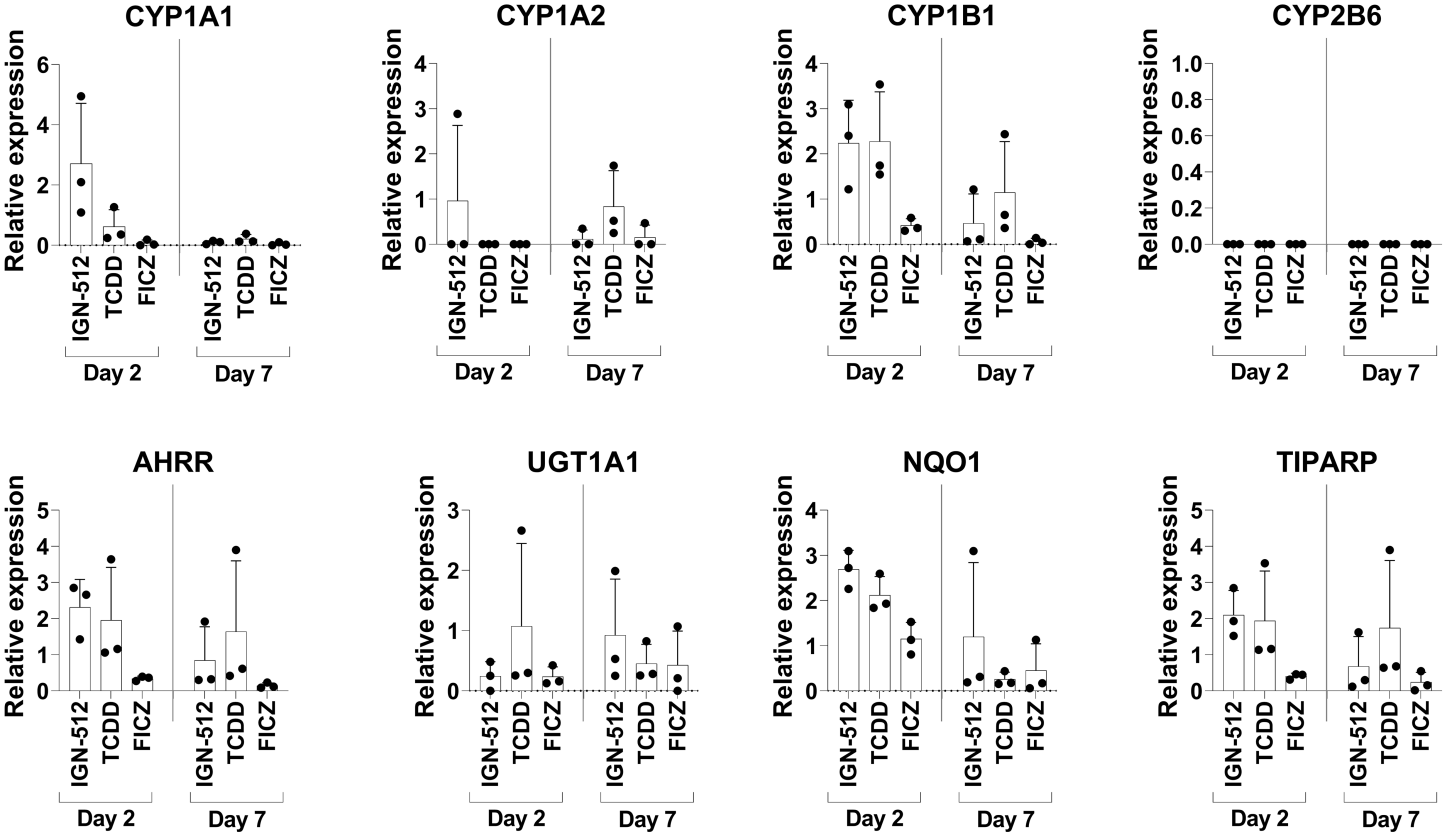
Gene expression of AhR activation-associated genes assessed on Day 2 in CD14+ cells treated with the AhR ligands IGN-512, FICZ or TCDD from start of the culture and assessed again on Day 7 after the cells were treated with AhR ligand and TGF-β on Day 3 and with RA on Day 6. RT-qPCR analysis of expression of the AhR activation-associated genes CYP1A1, CYP1A2, CYP1B1, CYP2B6, AHRR, UGT1A1, NQO1 and TIPARP assessed on Day 2 and Day 7 of the DC differentiation. Relative expression was calculated as 2^-ΔCT^ values (normalized to gene expression of the housekeeping genes: *B2M* and *RPL13a*). Data are presented as mean with SD where n=3.

To confirm that the AhR agonist IGN-512 activated AhR activation-associated genes, transcription of the *CYP1A1*, *CYP1A2*, *CYP1B1*, *CYP2B6*, *AHRR*, *UGT1A1*, *NQO1*, and *TIPARP* genes was assessed in cells treated with IGN-512. Gene expression was analyzed 48 h after IGN-512 was added and at Day 7, i.e. 96 h after the second addition of IGN-512. IGN-512-induced gene expression of AhR activation-associated genes was compared to the gene expression induced by TCDD and FICZ, two high-affinity AhR agonists. The results showed that ItolDCs treated with the AhR agonist IGN-512 upregulated mRNA expression of AhR-activation-associated genes (*CYP1A1, CYP1B1*, *AHRR*, *NQO1*, and *TIPARP*) equally well compared to TCDD on Day 2 of the DC differentiation (**Figure 2**). On Day 7, most of these upregulated genes (*CYP1A1*, *CYP1B1*, *AHRR*, *NQO1*, and *TIPARP*) were downregulated in IGN-512-treated cells (**Figure 2**). The cells treated with FICZ had a lower upregulation of these genes compared to cells treated with either IGN-512 or TCDD (**Figure 2**). The expression of the genes was also investigated in monocytes. The expression of CYP1B1, AHRR, and NQO1 was upregulated in IGN-512-treated cells on Day 2 compared to non-treated cells on Day 0 and Day 2 (**Supplementary Figure 7**). Indeed, IGN-512 activates AhR-associated genes like TCDD and FICZ do but with a distinct activation pattern.

### 3.3 ItolDCs display a weak pro-inflammatory cytokine profile

**Figure 3:**
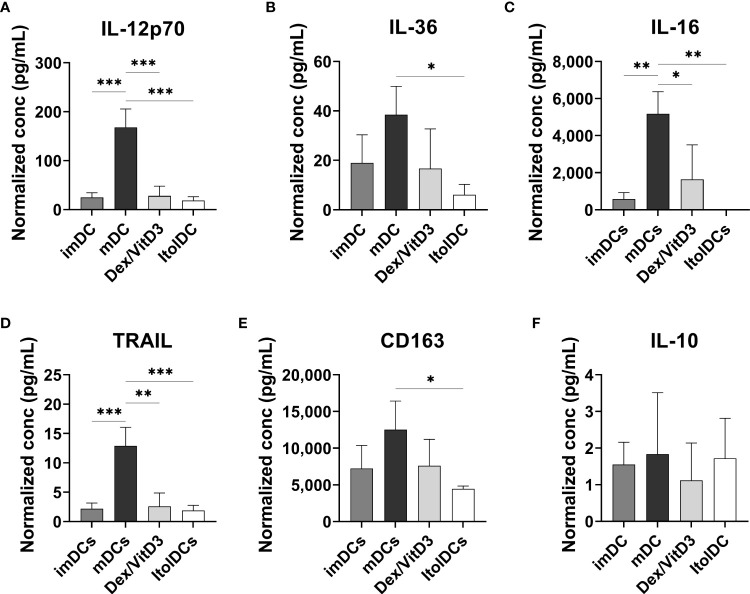
Assessment of cytokine secretion from DCs using multiplex assay. **(A-F)** Released IL-12p70, IL-36, IL-16, TRAIL, and CD163 determined in the supernatants from imDC, mDC (matured with TIP6 cocktail), Dex/VitD3-tolDC and ItolDC cultures after 7 days of differentiation. Cytokine concentrations were normalized to the number of cells in cultures. Cytokines were determined using magnetic bead-based multiplex assay. Data are presented as mean with SD where n=3, and one-way ANOVA with *post-hoc* testing was used for statistical comparisons. Statistical significances are presented as *=p<0.05, **=p<0.01, and ***=p<0.001.

The cytokine profiles of imDCs, mDCs (matured with TIP6 cocktail), Dex/VitD3-tolDCs, and ItolDCs were determined by assessing the cytokines released into the cell culture medium by the DCs during the 7-day-differentiation period. ItolDCs produced no or low levels of a wide range of different cytokines, both pro-inflammatory and anti-inflammatory, compared to mDCs (**Figures 3A–F** and **Supplementary Figures 8A–G**). Supernatants of mDC cultures showed high levels of TNF-α, IL-1β, and IL-6 as these cells were matured with a mixture of these cytokines (**Supplementary Figures 8A–C**). ItolDCs released lower levels of the pro-inflammatory cytokines IL-12p70, IL-36, and IL-16 compared to mDCs (**Figures 3A–C**). ItolDCs also released low levels of TNF-related apoptosis-inducing ligand (TRAIL) and CD163 compared to mDCs (**Figures 3D, E**). The data showed that ItolDCs do not have a pro-inflammatory cytokine profile and that the concentration of IL-10 released from ItolDCs was very low (**Figure 3F**).

### 3.4 ItolDCs induce less T cell proliferation and increased the proportions of regulatory T and B cells

**Figure 4:**
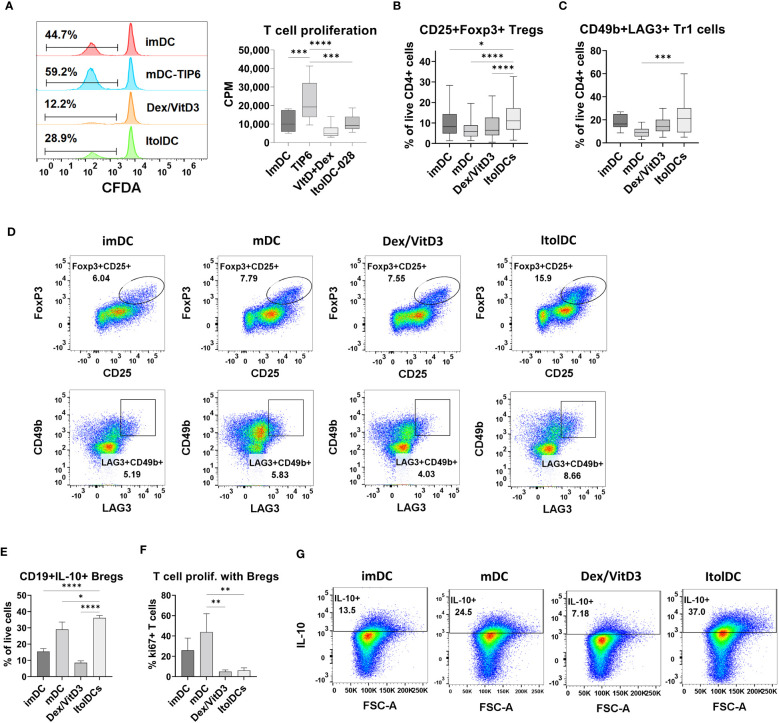
Functional assessment of ItolDCs and control DCs (imDCs, mDCs matured with TIP6 cocktail, and Dex/VitD3-tolDCs) were assessed in MLR. **(A)** Induced T cell proliferation determined by CFDA dilution and incorporation of [^3^H]-thymidine, and frequencies of **(B)** CD25+Foxp3+ Tregs of CD4+ T cells and **(C)** CD49b+LAG3+ Tr1 cells of CD4+ T cells were assessed by flow cytometry. **(D)** Representative plots for gated Treg and Tr1 cells after culture with DCs. **(E)** Induction of IL-10-producing Bregs was determined in autologous co-cultures of DCs and B cells, and IL-10 was detected intracellularly using flow cytometry. **(F)** T cell proliferation induced in autologous co-cultures of DCs, B cells and T cells was determined using flow cytometry. **(G)** Representative plots for IL-10+ Bregs after co-culture with DCs. Data are presented as mean with SD (A with n=12, B with n=65-80, C with n=10-18, and E-F with n=3), and one-way ANOVA with *post-hoc* testing was used for statistical comparisons. Statistical significances are presented as *=p<0.05, **=p<0.01, ***=p<0.001, and ****=p<0.0001.

The capacity of ItolDCs and of control DCs to activate T cells was investigated in MLR by quantifying T cell proliferation. ItolDCs induced less T cell proliferation compared to mDCs but induced comparable proliferation to that induced by imDCs and Dex/VitD3-tolDCs (**Figure 4A**). Furthermore, the capacity of ItolDCs to increase the proportions of regulatory T cells, both CD25+Foxp3+ cells and CD49b+LAG3+ Tr1 cells, in cultures was assessed in the MLR. ItolDC-cultures with T cells contained significantly higher proportions of regulatory T cells of both types compared to mDC-cultures (**Figures 4B–D** and **Supplementary Figure 9A**). Moreover, the capacity of ItolDCs to induce Bregs was studied in co-cultures of autologous DCs with both , CD19+ B cells and T cells in the presence of the B cell activator CpG-ODN (**Figures 4E–G**). The results showed that co-cultures with ItolDCs had a significantly higher frequency of CD19+IL-10+ Bregs compared to co-cultures with the control DCs (**Figure 4E**). Even though mDC-cultures had a high frequency of CD19+IL-10+ Bregs (**Figure 4E**), the induced T cell proliferation in co-cultures with ItolDCs was on average at least 5 times lower compared to co-cultures with mDCs (**Figure 4F**). Taken together, these data showed that ItolDCs in *in-vitro* cultures induced less T cell proliferation compared to mDCs and induced a larger proportion of regulatory T and B cell in the cultures than imDCs, mDCs, and Dex/VitD3-tolDCs.

### 3.5 Assessment of intracellular cytokines in T cells cultured with ItolDCs showed a less activated and more anti-inflammatory profile

**Figure 5:**
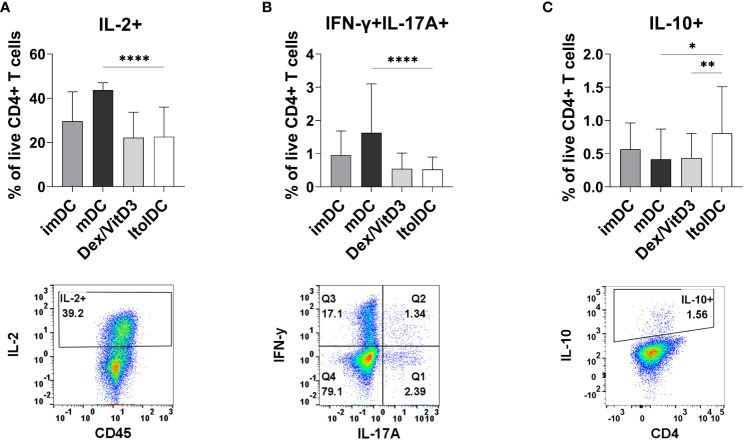
Detection of intracellular T cell cytokines **(A)** IL-2, **(B)** IFN-ɣ and IL-17A, and **(C)** IL-10 were determined intracellularly after 7 days of MLR with DCs. Figure shows cytokine and respective gating strategy. Cultures were on Day 7 stimulated for 5 h with PMA/ionomycin, and monensin were added to the cultures during the last 2 h. Frequencies of IL-2+, IFN-ɣ+IL-17A+, and IL-10+ of viable CD4+ T cells were assessed using flow cytometry. Intracellular cytokines of T cells co-cultured with ItolDC were compared to the cytokines from T cells co-cultured with control DCs (imDCs, mDCs, and Dex/VitD3). Data are presented as mean with SD where n=12-39, and one-way ANOVA with *post-hoc* testing was used for statistical comparisons. Statistical significances are presented as *=p<0.05, **=p<0.01, and ****=p<0.0001.

After seven days of culture, the intracellular cytokine profile of T cells cultured with allogeneic ItolDCs was determined. T cells were activated with PMA and ionomycin and thereafter treated with monensin to assess the levels of intracellular IL-2, IFN-ɣ, IL-17A, and IL-10. The frequency of intracellular IL-2 in T cells and in T cells co-expressing IFN-ɣ and IL-17A were both significantly lower when cultured with ItolDCs compared to with mDCs and equal to when cultured with Dex/VitD3-tolDCs (**Figures 5A, B** and **Supplementary Figure 9B**). Moreover, the frequency of T cells with intracellular IL-10 was significantly higher in T cells cultured with ItolDCs compared to in T cells cultured with mDCs and Dex/VitD3-tolDCs (**Figure 5C**). These data showed that T cells in MLRs with ItolDCs compared to mDCs have fewer cells expressing pro-inflammatory cytokines and more cells expressing anti-inflammatory cytokines.

### 3.6 ItolDCs present loaded antigen on HLA-DR and antigen loading did not affect the phenotype or function of ItolDCs

**Figure 6:**
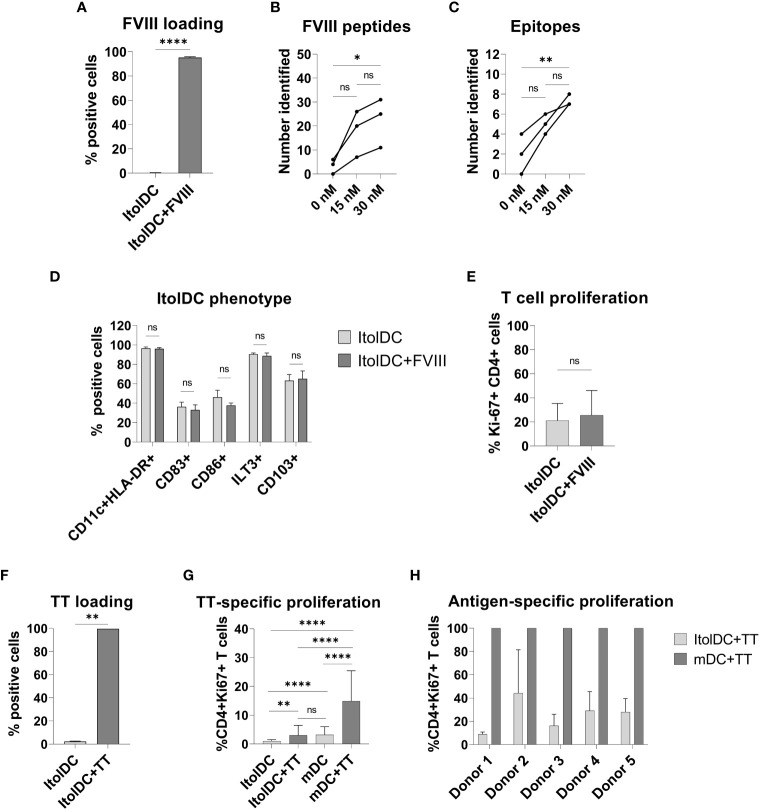
Phenotype and functional assessment after antigen loading of ItolDCs. **(A)** Frequency of cells that were positive for intracellular FVIII after loading was assessed using flow cytometry. **(B, C)** Number of identified FVIII peptides and epitopes after mass spectrometric analysis of HLA-DR-bound FVIII peptides of ItolDCs loaded with 15 or 30 nM FVIII. **(D)** Phenotype of ItolDCs loaded with FVIII was determined based on the frequencies of CD11c+HLA-DR+, CD83+, CD86+, ILT3+ and CD103+ cells of live cells and compared to ItolDCs not loaded with FVIII. **(E)** T cell proliferation in an MLR was determined for ItolDCs and ItolDCs loaded with FVIII. **(F)** Frequency of cells that were positive for AF488-labelled TT after loading on Day 3 was assessed on Day 7 using flow cytometry. **(G)** Antigen-specific T cell proliferation of TT-responsive donors was determined using ItolDCs and mDCs (matured with NIH low cocktail) where both were loaded with 30 nM TT on Day 6 and thereafter co-cultured for 7 days with autologous CD4+ T cells from Day 7. CD4+ T cells were co-cultured with either ItolDCs or mDCs with six replicates for each condition. T cell proliferation was determined by assessment of frequency of Ki-67+ of CD4+ cells using flow cytometry. **(H)** The T cell proliferation of individual donors from Figure E where TT-specific response was presented as relative percentage of induced proliferation compared to mDCs loaded with TT. Data are presented as mean with SD (A and D with n=4, B and C with n=3, and E and F with n=2, and G with n=5, and H with n=6 replicates). Either t-test or one-way ANOVA with *post-hoc* testing was used for statistical comparisons. Statistical significances are presented as ns=non-significant, *=p<0.05, **=p<0.01, and ****=p<0.0001.

**Figure 7:**
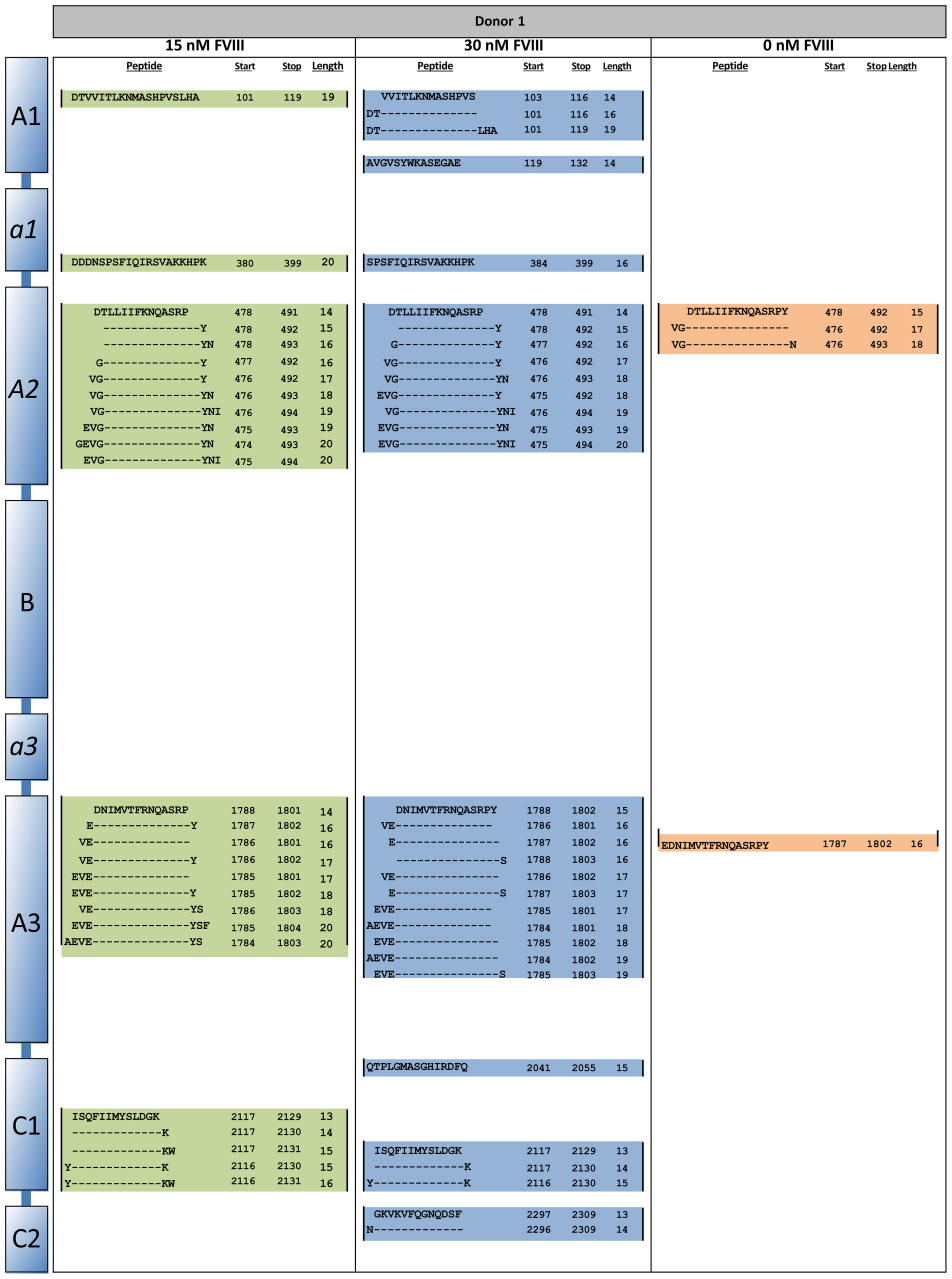
HLA-DR peptide profiling of FVIII-loaded ItolDCs. ItolDCs were loaded with 15 or 30 nM FVIII and both were compared to not loaded ItolDCs. To assess antigen processing and presentation, ItolDCs were lyzed and eluted peptides were identified using mass spectrometry and Proteome Discoverer 1.4. Figure shows one representative donor out of three donors.

To study antigen-uptake and presentation, ItolDCs were loaded with FVIII, the antigen that will be used for induction of tolerance in Hemophilia A patients who have developed neutralizing antibodies against FVIII. FVIII uptake was detected by intracellular staining using an anti-FVIII antibody labeled with AF488 and flow cytometry. ItolDCs showed >95% uptake of FVIII 24 hours after addition of the antigen (**Figure 6A**). To assess uptake of FVIII by ItolDCs, both the extracellular and intracellular presence of FVIII was investigated and showed low frequencies (>5%) of extracellular FVIII (**Supplementary Figure 10**). To study antigen uptake and the ability of ItolDCs to process and present FVIII peptides on HLA-DR molecules, a mass spectrometric analysis was performed on peptides released from HLA-DR molecules that was affinity-purified from lyzed cells. Data showed that FVIII peptides were identified in all FVIII-loaded donor samples over the A1, a1, A2, A3, C1, and C2 domains of FVIII (**Figures 6B, C** and **Figure 7**). When cells were loaded with higher concentrations of FVIII, more peptides were identified (**Figure 7**), suggesting that antigen-loaded ItolDCs were able to process and present FVIII peptides on HLA-DR molecules. The phenotype and function of ItolDCs with and without loaded antigen were compared. Data showed that cells loaded with FVIII were not different in their phenotype nor in their ability to stimulate T cell proliferation in an MLR compared to cells not loaded with antigen (**Figures 6D, E**).

Moreover, the antigen-specific T cell response induced by ItolDCs was investigated using TT-loaded ItolDCs. TT was used as a model antigen as most blood donors have TT-responsive memory T cells. By loading ItolDCs with AF488-labeled TT, antigen uptake was determined using flow cytometry. Data showed that ItolDCs were >99% positive for TT-AF488 four days after adding the antigen (**Figure 6F**). TT-specific T cell proliferation induced by TT-loaded ItolDCs and TT-loaded mDCs was assessed in autologous co-cultures. ItolDCs and mDCs not loaded with TT were used as controls for unspecific background proliferation. Data showed that there was a low TT-specific proliferation of T cells when ItolDCs were loaded with TT, but this increase was however not higher than the background proliferation induced by mDCs without TT loading (**Figure 6G**). When mDCs were loaded with TT, a high antigen-specific T cell proliferation was observed in each donor compared to the proliferation induced by respective donor of TT-loaded ItolDCs (**Figures 6G, H**). Collectively, these data showed that antigen loading on ItolDCs has no impact on the phenotype of the cells and that antigen-specific T cell responses can be induced.

### 3.7 ItolDCs retain a stable tolerogenic phenotype after pro-inflammatory challenge *in vitro*

**Figure 8:**
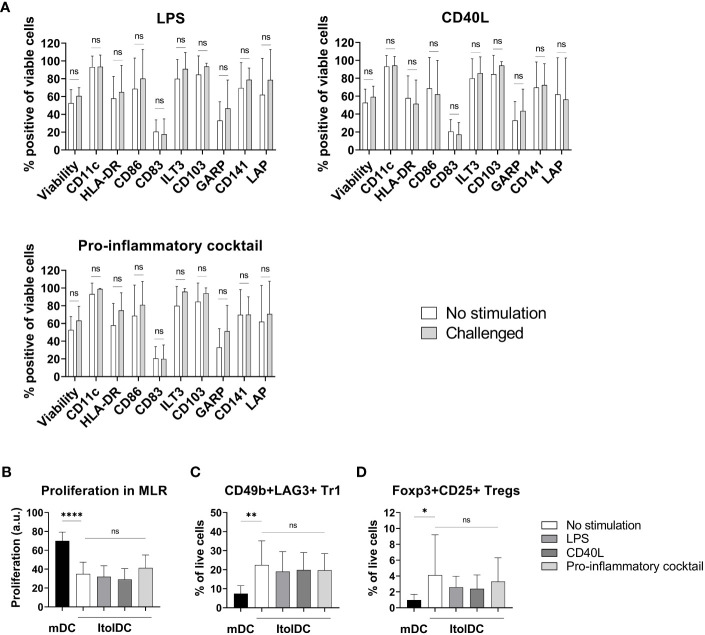
Phenotypic and functional assessment of ItolDCs after pro-inflammatory challenge of cryopreserved and thawed DCs. **(A)** A comparison of the phenotype of non-challenged ItolDCs to the phenotype of ItolDCs upon challenge with physiological levels of LPS, CD40L, or a pro-inflammatory cocktail consisting of IFNα, IL-6, IL-1β, TNFα, and IL-8, determined as frequencies of CD11c, HLA-DR, CD86, CD83, ILT3, CD103, GARP, CD141 and LAP. **(B)** Induction of T cell proliferation, and **(C)** frequencies of Tr1 cells and **(D)** CD25+Foxp3+ Tregs by DCs after pro-inflammatory challenge of ItolDCs. Proliferation was assessed by CFDA dilution determined by flow cytometry. Data are presented as mean with SD (A with n=7-10, B with n=4-14, and C-D with n=10-20), and one-way ANOVA with *post-hoc* testing was used for statistical comparison. Statistical significances are presented as ns=non-significant, *=p<0.05, **=p<0.01, and ****=p<0.0001.

**Figure 9:**
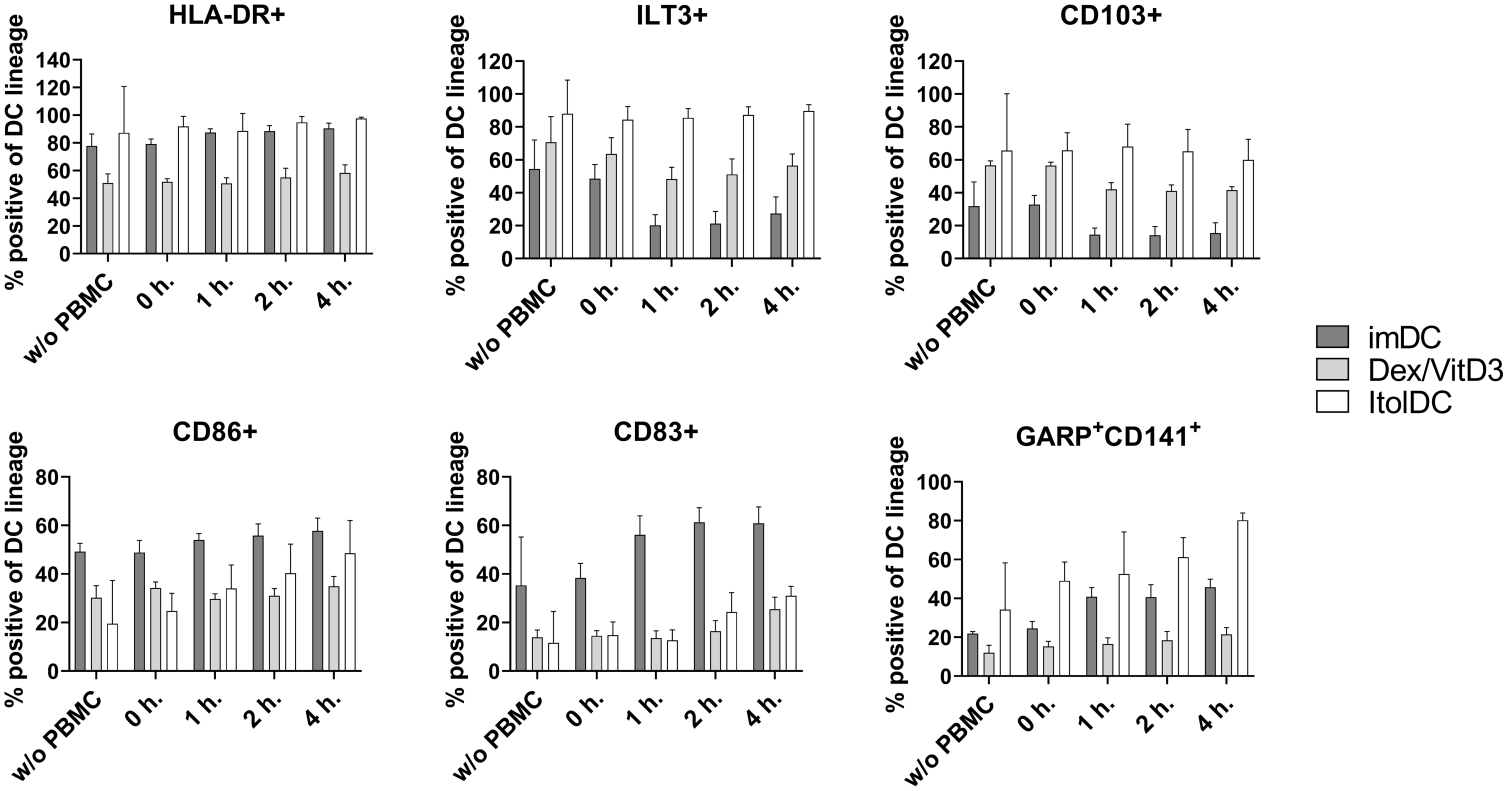
Phenotypic assessment of ItolDCs upon co-culture with allogeneic PBMCs was determined based on the frequencies of HLA-DR+, ILT3+, CD103+, CD86+, CD83+, CD141+GARP+ of live ItolDCs before co-culture with PBMCs, and at 0, 1, 2 and 4 hours in allogeneic co-culture with PBMCs. Data are presented as mean with SD (n=3-6), and one-way ANOVA with *post-hoc* testing was used for statistical comparison.

To assess the stability of ItolDCs’ tolerance-inducing phenotype in an inflammatory environment, such as in an unidentified infection, they were subjected to various stimulations. ItolDCs were resistant to maturation after stimulation with LPS, with the adaptive immune cell co-stimulatory molecule CD40L, or with a mixture of pro-inflammatory cytokines (IFNα, IL-6, IL-1β, TNFα, and IL-8) (**Figure 8A**). Compared to non-stimulated cells, challenged ItolDCs showed no changes in viability, phenotype, or function, determined as capacity to induce T cell proliferation and Tregs (**Figures 8A–D**). Interaction with other immune cells and their microenvironment may also influence the phenotype of DCs. To study this, allogenic PBMCs were cultured with ItolDCs, and the phenotype of ItolDCs was analyzed after 1, 2, and 4 h of co-culture. Data showed that expression of HLA-DR, ILT3, and CD103 was stable for ItolDCs during the indicated period, while the expression of CD86, of CD83, and of cells co-expressing GARP and CD141 was slightly increased over time (**Figure 9**). Altogether, stability tests *in vitro* showed that ItolDCs were stable, and when challenged with physiologically relevant inflammatory stimuli, the challenges did not negatively impact ItolDCs’ viability, phenotype, homogeneity, or potency.

## 4 Discussion

The results of the investigations presented here show that phenotypically and functionally stable tolDCs can be generated *in vitro* by differentiation of CD14+ monocytes using a novel combination of three compounds, specifically IGN-512, TGF-β, and RA, that synergistically act to generate a stable tolerogenic profile of the DCs. The ItolDCs induced with this combination of compounds have the potential to constitute the base of a tolDC platform technology for inducing antigen-specific tolerance in disorders caused by unwanted immune cell activation.

Throughout the studies presented here, phenotype and function of ItolDCs were compared to imDCs, mDCs, and Dex/VitD3-tolDCs. Different variants of Dex/VitD3-tolDCs have previously been used in clinical studies for tolerance induction (28). It is evident that ItolDCs share phenotypic characteristics with Dex/VitD3-tolDCs, such as low expression levels of HLA-DR, CD80, CD83, and CD86 molecules. In contrast, ItolDCs express higher densities (higher MFI values) of the tolerance-associated molecules ILT3, CD103, and LAP, and a higher frequency of GARP+CD141+ cells compared to Dex/VitD3-tolDCs. A study showed that DCs generated from monocytes using RA have an increased expression of CD103, and inhibition of TGF-β signaling downregulates the CD103 expression (35). RA upregulate both CD141 and GARP on DCs (4), and as GARP is a receptor for latent TGF-β, both LAP and mature TGF-β can bind to it (36). The tolerance-associated molecule IDO was not expressed in ItolDCs, nor in Dex/VitD3-tolDCs. IDO was however interestingly found expressed in mDCs matured with TIP6 but not in mDCs matured with LPS. Lanzinger et al. showed that PGE2 in the TIP6 cocktail was crucial for IDO expression while TNF-α was required to effectively induce IDO competence (37, 38), which explains the expression of IDO in the TIP6-matured mDCs. Although another study showed that IDO expression can be induced using LPS, it required the presence of IFN-ɣ (39).

The development of ItolDC was initiated by investigating the tolerance-inducing capacity of IGN-512, TGF-β, and RA, separately and in different combinations. When used separately, the generated tolDCs did not obtain a strong tolerogenic phenotype or functionality. Activation of AhR with IGN-512 showed signs of a tolerogenic phenotype but exhibited at the same time inflammatory functions when stimulated with LPS by producing on average 15% higher IL-23 than LPS-stimulated imDC. This increased production of IL-23 was reduced by 40-50% when IGN-512 was combined with TGF-β or RA. IL-23 promotes effector T cells that secretes other proinflammatory cytokines such as IL-17A, IL-17F, IL-6, and TNF-α. Thus, a lower production of IL-23 would benefit the tolerogenic functions of ItolDCs (40). Both TGF-β and RA have previously been described as important mediators in establishing a tolerogenic environment during the development of tolDCs and other regulatory immune cells (41). Changing the context of AhR activation by combining IGN-512 with RA or TGF-β resulted in a more tolerogenic profile of the cells, demonstrating that induction of tolDCs using IGN-512 requires involvement of other compounds to generate cells with a strong tolerogenic phenotype and function. RA and AhR agonists have previously been shown to influence cellular targets in a synergistic manner and that AhR signaling enhances RA-induced cell activities (42, 43). In addition, DCs generated with IGN-512 had lower expression of ILT3 compared to when DCs were generated in combination with TGF-β and RA. Also, co-presence of TGF-β can modulate the resulting downstream effects, such as NF-kB activation, initiated by AhR activation. These findings confirm the dual role of AhR activation in terms of downstream effector functions and emphasizes the importance of the context during which AhR activation occurs.

Moreover, different types of AhR ligands may exert distinct effects after interaction with their receptor as DCs differentiated in the presence of different AhR ligands impacted their phenotype and/or function differently (24, 44–47). For instance, in contrast to DCs treated with the AhR agonists FICZ or ITE (27), IGN-512-treated DCs stimulated with LPS produced high amounts of IL-23 and no IL-10. However, the use of different starting material (cells from Behçet’s disease patient vs. healthy donors) could potentially have been impacting the results of this investigation. AhR agonists may differentially activate AhR which was reflected in the results from the gene expression analysis of cells treated with IGN-512, TCDD, or FICZ. The data confirms that the synthetic AhR agonist IGN-512 activates genes that are also affected by other AhR agonists but with a different gene expression profile compared to that of TCDD and FICZ. CYP1A1, CYP1A2, CYP1B1, NQO1, and ALDH3A1 are the main AhR-affected genes, all encoding xenobiotic-metabolizing enzymes, including the cytochrome P450 family members A1, A2 and B1 (48).

The main aim of a tolDC therapy is to induce immune cells with tolerogenic functions, for example regulatory T and B cells (49–51). A key mechanism by which tolDCs restore peripheral tolerance is via induction of Tregs that can counteract both pathological T- and B-cell responses. TolDCs also have the ability of initiating a feedback loop where tolDCs polarize naïve T cells into Tregs and, in turn, Tregs secrete anti-inflammatory cytokines that influence DCs and other immune cells (52). The studies presented here showed that ItolDCs have the capacity to increase the proportions of Tregs, both CD25+Foxp3+ Tregs and CD49b+LAG3+ Tr1 cells, and IL-10-producing Bregs. Due to donor variations and different degree of HLA-mismatch between the donors in the MLR assays, the percentage of induced regulatory cells and proliferation of T cells in ItolDC-cultures vary. To reduce the biological inter-variability in proliferation assays, Nicotra et al. pooled PBMCs of 10 donors to create an MLR assay that was validated according to ICH Q2 standard (53). The higher expression of tolerance-associated markers on ItolDCs compared to Dex/VitD3-tolDCs, and difference in frequencies of regulatory cells induced, could possibly mean that ItolDCs induce regulatory immune cells via other pathways compared to those employed by Dex/VitD3-tolDCs. A hypothesis which will be further investigated. It has been shown that CD103+ intestinal DCs are dependent on TGF-β and RA for generation of Tregs from naïve CD4+ T cells (15, 16). Coombes et al. showed that Foxp3+ Treg induction was completely absent when TGF-β was blocked by neutralizing antibodies and that only CD103+, but not CD103-, DCs had the capability to induce Foxp3+ Tregs. CD103+ DCs express *aldh1a2*, a retinal dehydrogenase involved in the conversion of retinal into RA, whereas CD103- DCs lack expression of this enzyme (16). RA promotes the expression of CTLA-4 on Foxp3+ Tregs, enhances CD103 expression in T cells, and is a key regulator of TGF-β-dependent immune responses (54). Foxp3+ Tregs differentiated *in vitro* using RA together with TGF-β were able to suppress inflammation upon transfer *in vivo* in RAG-1^-/-^ mice (54).

Differentiation of Tr1 cells is known to be driven by IL-10, and Tr1 cells are known for producing IL-10. However, despite not secreting detectable levels of IL-10, CD49b+LAG3+ Tr1 cells are induced when cultured with ItolDCs. It has previously been shown that RA can induce autoantigen-specific Tr1 cells (55), and as ItolDCs are generated using RA, this may be the underlying mechanism in Tr1 induction. AhR is activated in Tr1 cells during Tr1 induction, and TGF-β is suggested to have a role in Tr1 induction (56). As ItolDCs are generated with both an AhR agonist and TGF-β, it may also contribute to the induction of Tr1 cells. IL-10 signaling is needed to maintain IL-10 production in Tr1 cells. Even though IL-10 production is impaired in the absence of this signaling, Tr1 cells may use other alternative mechanisms to induce suppression as they can still express Granzyme B and CTLA-4 (57, 58). This could be an explanation to why there is a discrepancy between the frequency of Tr1 cells in ItolDC-cultures and the few percentages of CD4+ T cells that are positive for intracellular IL-10. In further studies, it will be necessary and important to functionally characterize the different potentially suppressive subsets (Tr1 cells, Foxp3 Tregs, and Bregs) induced by ItolDCs to gain a better understanding of the *in vivo* functionality of ItolDC.

The detection of HLA-DR-bound FVIII peptides verified that ItolDCs process antigens after uptake and present the processed peptides on HLA-DR to other immune cells (59). When antigen-presenting cells are able to process antigens for antigen-presentation, the repertoire of relevant immune-stimulating FVIII peptides may be more optimal than if the cells were fed with selected or overlapping peptides (59). Although FVIII-specific naïve and memory CD4+ T cells can be found in healthy individuals, these cells do not expand upon encountering FVIII (60). Therefore, TT was used to study antigen-specific responses induced by ItolDCs. Consistent with what has previously been described for other tolDCs, the functional studies with antigen-loaded DCs showed that TT-loaded ItolDCs could, compared to non-loaded ItolDCs, induce a limited T cell proliferation in autologous co-cultures (8, 28). This data supports the potential use of ItolDCs as a platform technology to induce antigen-specific T cell suppression. With the ItolDC-platform, different antigens can be loaded onto the cells to induce immunological tolerance towards the specific antigen or antigens causing the disease.

A prerequisite for a cell therapy based on tolDCs is that the cells remain tolerogenic in inflammatory environments. To verify that ItolDCs were stable, they were challenged *in vitro* with LPS, CD40L, or with a mixture of inflammatory cytokines. The data showed that ItolDCs were both phenotypically and functionally stable, which supports the notion that the cells will not change into DCs with immune activating potential when administered clinically. Our data also support that interaction with other immune cells does not significantly impact the phenotype of ItolDCs.

Even though AhR agonists, TGF-β, and RA are naturally present at mucosal sites and contributes to tolerance induction, injecting the combination of IGN-512, TGF-b, and RA systemically is not considered a viable option because of the risk of toxicity as the concentrations used for the generation of ItolDCs are higher than what if found in normal serum levels (61, 62). Further, it will be very difficult to accomplish antigen-specific tolerance following systemic injection of the antigen, unless the compounds are encapsulated (63), e.g. packed into microvesicles that are specifically targeted to DC. Thus, induction of tolDCs and antigen-loading *ex vivo* followed by administration of the cells seems to be a more realistic approach to accomplish antigen-specific immune tolerance.

In conclusion, the data presented here show that a novel method to generate tolDCs *in vitro* with the capacity to increase the frequencies of both regulatory T and B cells has been developed. The results presented herein show that the addition of an antigen to the process of generating ItolDCs did not impact the defined tolerogenic phenotype of these cells, and the data showed that ItolDCs can process and present FVIII on their HLA-DR molecules after antigen loading. Moreover, ItolDCs loaded with TT increased TT-specific T cell proliferation which suggests that an antigen-specific response could also be induced by ItolDCs. In the first planned clinical trial using the ItolDC platform, generated cells will be loaded with FVIII and administered to Hemophilia A patients with neutralizing antibodies to FVIII. The aim with this treatment is to stop the unwanted immune activation against FVIII by inducing FVIII-specific tolerance such that these patients can resume their standard FVIII-replacement treatment.

## Data availability statement

The data presented in the study are deposited in the PRIDE repository, accession number PXD045810.

## Ethics statement

Leukocyte concentrates of healthy donors were purchased from the local blood bank at Skåne university hospital, department of Clinical Immunology and Transfusion Medicine, Lund, Sweden with the approval of the Swedish Ethical Review Authority (Etikprövningsmyndigheten).

## Author contributions

GD, LP, PE, TH, AS, DA, KT, HR, and MW planned the experiments. GD, LP, PE, TH, KT, and MW performed the experiments, analyzed and interpreted the data. GD, KT, and MW wrote the manuscript. LO reviewed the manuscript. All authors contributed to the article and approved the submitted version.
