# Supplementary material for: Tolerogenic dendritic cells generated in vitro using a novel protocol mimicking mucosal tolerance mechanisms represent a potential therapeutic cell platform for induction of immune tolerance
Source: Front Immunol. 2023 Oct 13;14:1045183. doi: 10.3389/fimmu.2023.1045183 (PMC10613069; doi:10.3389/fimmu.2023.1045183)
Supplement: Supplementary file 1 [file Presentation_1.pdf]

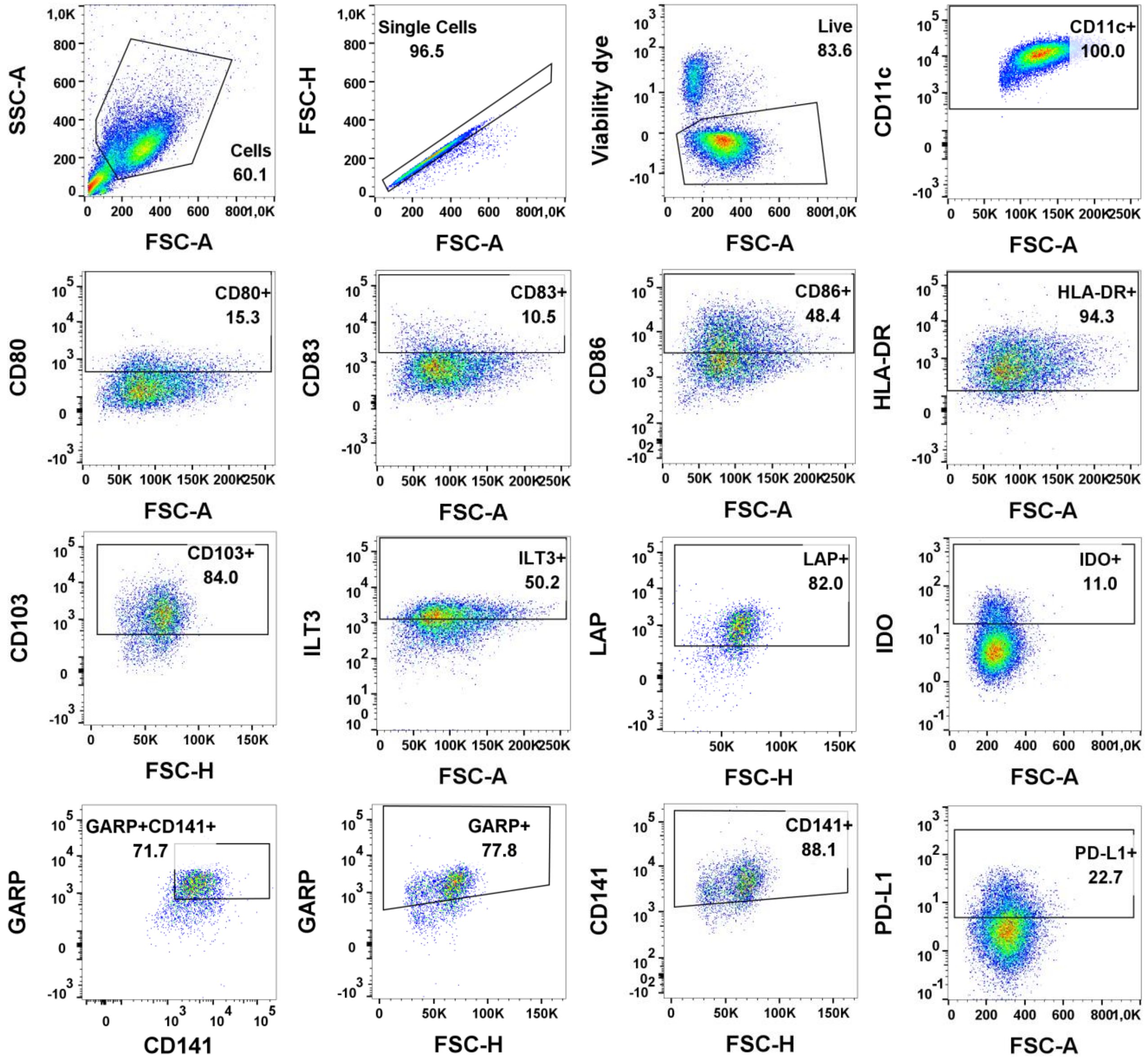

Supplementary Figure 1. Gating strategy for the flow cytometric assessment of dendritic cells. All the markers were gated from the live gate and according to controls. MFI levels were assessed on live cells. Representative plots for ItolDC.

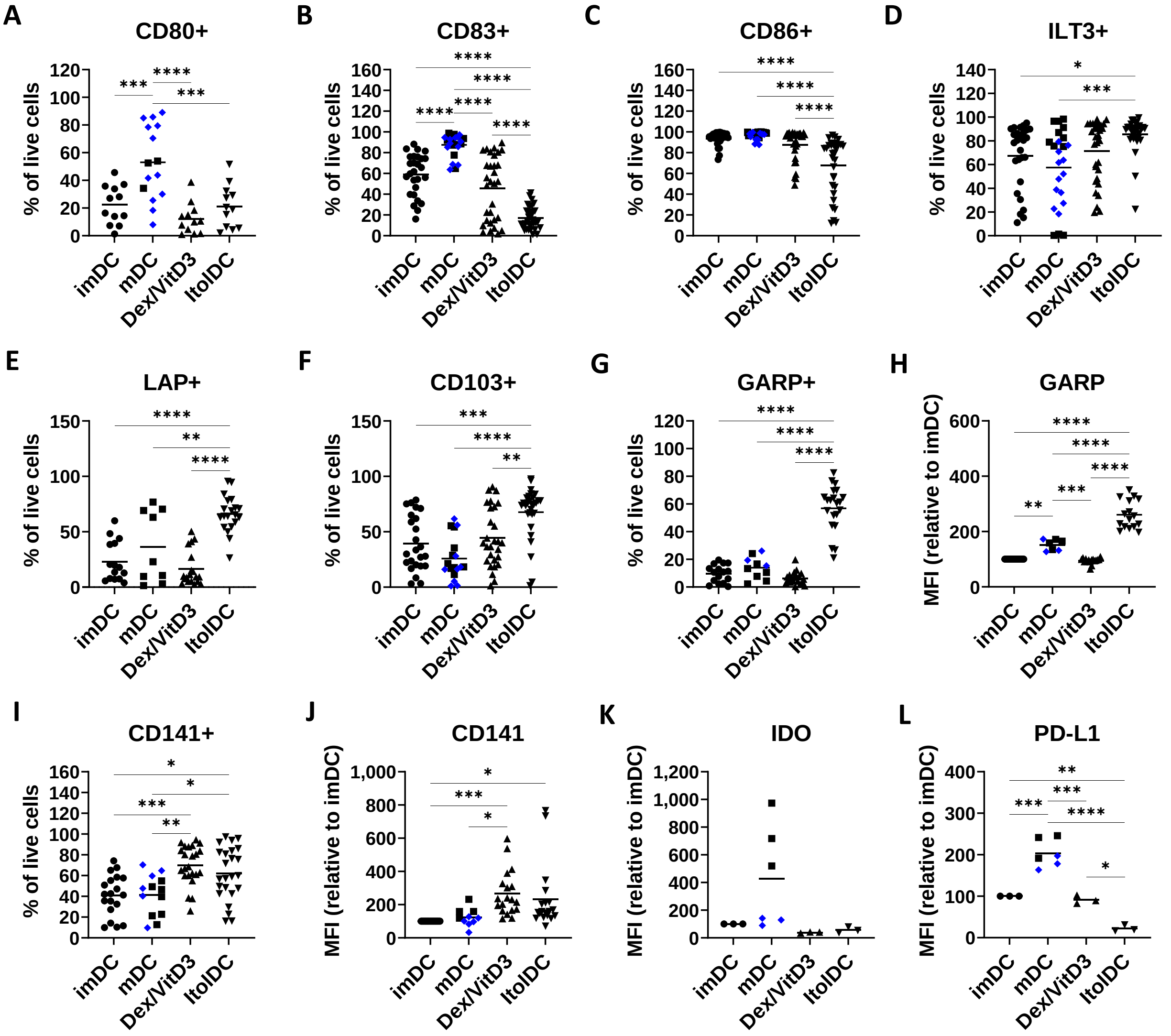

Supplementary Figure 2. ItolDC phenotype assessed using flow cytometry in comparison to imDCs, mDCs matured with either LPS (blue diamond, ♦) or TIP6 cocktail (black square, ■) and Dex/VitD3-tolDCs. **(A-F)** ItolDC phenotype assessed using flow cytometry and frequencies of CD80+, CD83+, CD86+, ILT3+, LAP+, and CD103+ cells. **(G-J)** Frequencies of GARP+ and CD141+ cells, and MFI levels of GARP and CD141. **(K-L)** MFI levels of IDO and PD-L. MFI values were calculated as relative to imDC, where MFI levels of imDC for each donor was set to 100. Frequencies were determined on live cells and data are presented as individual values with mean with SD (A with n=12-15, B-C with n= 24-32, D with n=21-32, E with n=9-20, F with n=18-29, G with n=10-21, H with n=7-16, I with n=13-24, J with n=10-19, and K-L with n=3-6. One-way ANOVA with post-hoc testing was used for statistical comparison. Statistical significances are presented as *=p<0.05, **=p<0.01, ***=p<0.001, and ****=p<0.0001.

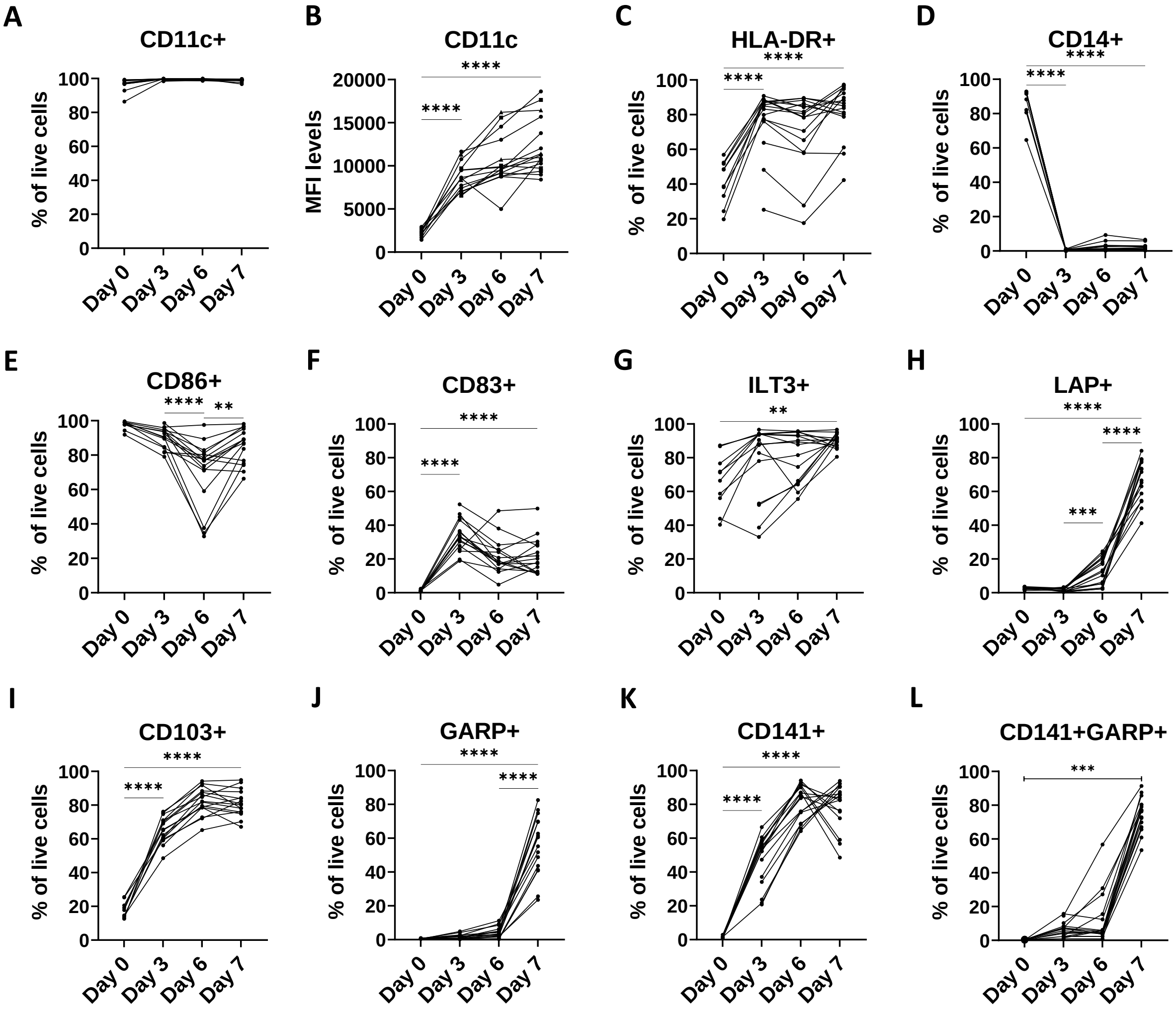

Supplementary Figure 3. Phenotypic characterization of ItolDCs during differentiation from CD14+ monocytes. **(A)** Frequency of CD11c-positive cells and **(B)** MFI of CD11c, **(C)** frequency of cells positive for HLA-DR**, (D)** CD14 **(E)** CD83, **(F)** ILT3, **(G)** LAP, **(H)** CD86, **(I)** CD103, **(J)** GARP, or **(K)** CD141, and **(L)** frequency of CD141 and GARP double-positive cells, assessed at Day 0, 3, 6 and 7 of differentiation from CD14+ monocytes to ItolDCs. Frequencies and MFI values were determined on live cells and data are presented as individual values where n=10-16, and one-way ANOVA with post-hoc testing was used for statistical comparisons. Statistical significances are presented as **=p<0.01, ***=p<0.001, and ****=p<0.0001.

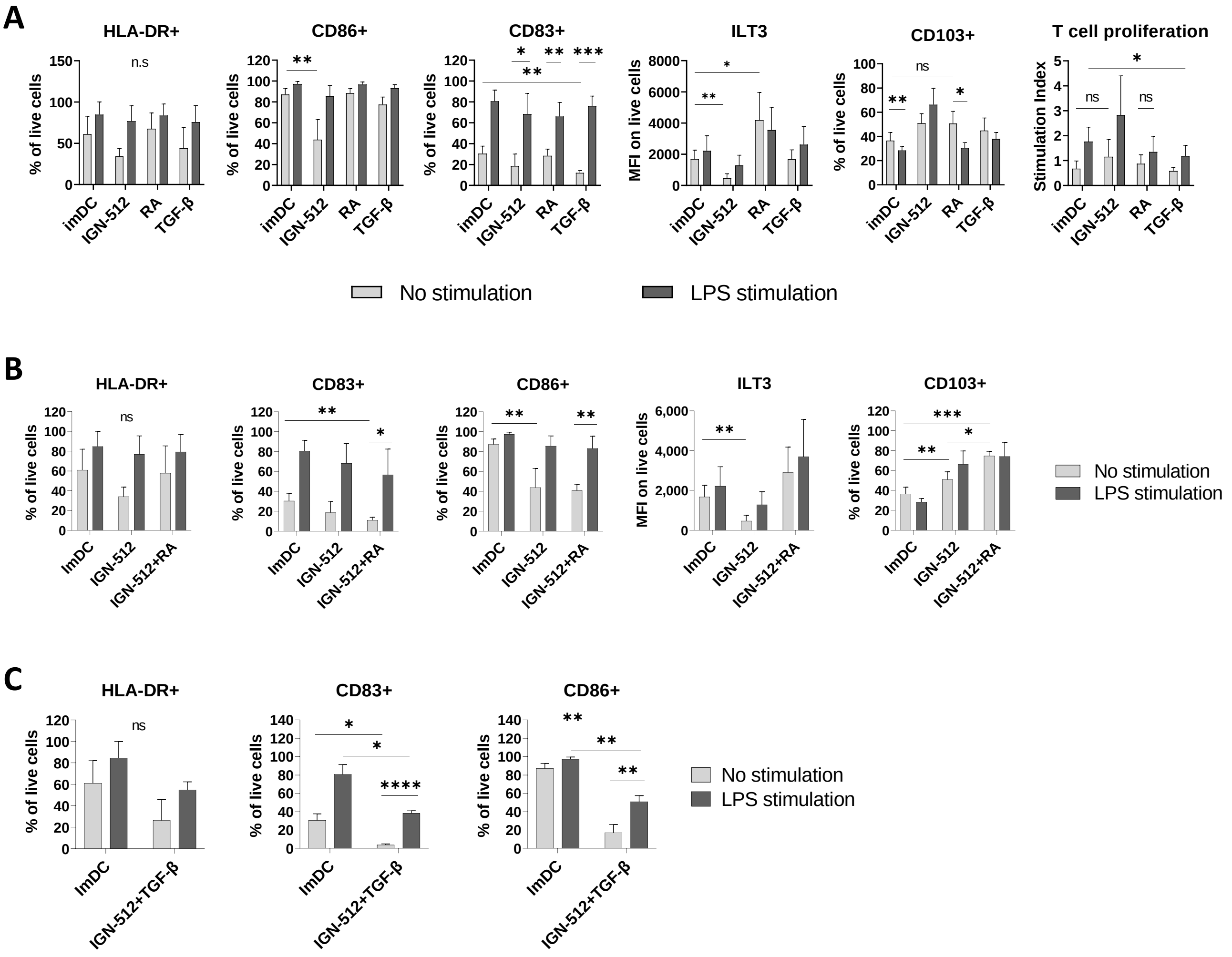

Supplementary Figure 4. Phenotypic and functional assessment of DCs differentiated in the presence of IGN-512, retinoic acid (RA) and/or TGF-β using flow cytometry. **(A)** Frequencies of HLA-DR+, CD83+, CD86+ and CD103+ cells of live cells and MFI levels of ILT3 on live cells are presented for imDCs and DCs differentiated in the presence of IGN-512, RA or TGF-β without and with LPS stimulation to induce maturation. Induction of T cell proliferation in an MLR with CD14-depleted PBMCs and with imDCs and DCs differentiated in the presence of IGN-512, RA or TGF-β. **(B)** Frequencies of HLA-DR+, CD83+, CD86+ and CD103+ of live cells and MFI levels of ILT3 on live cells are presented for imDCs and DCs differentiated in the presence of IGN-512 or IGN-512+RA without and with LPS stimulation to induce maturation. **(C)** Frequencies of HLA-DR+, CD83+ and CD86+ cells of live cells are presented for imDCs and DCs differentiated in the presence of IGN-512+TGF-β without and with LPS stimulation to induce maturation. Data are presented as mean with SD (A with n=3-8, and B-C with n=3-4), and t-test was used for statistical comparisons. Statistical significances are presented as ns=non-significant, *=p<0.05, **=p<0.01, ***=p<0.001, and ****=p<0.0001.

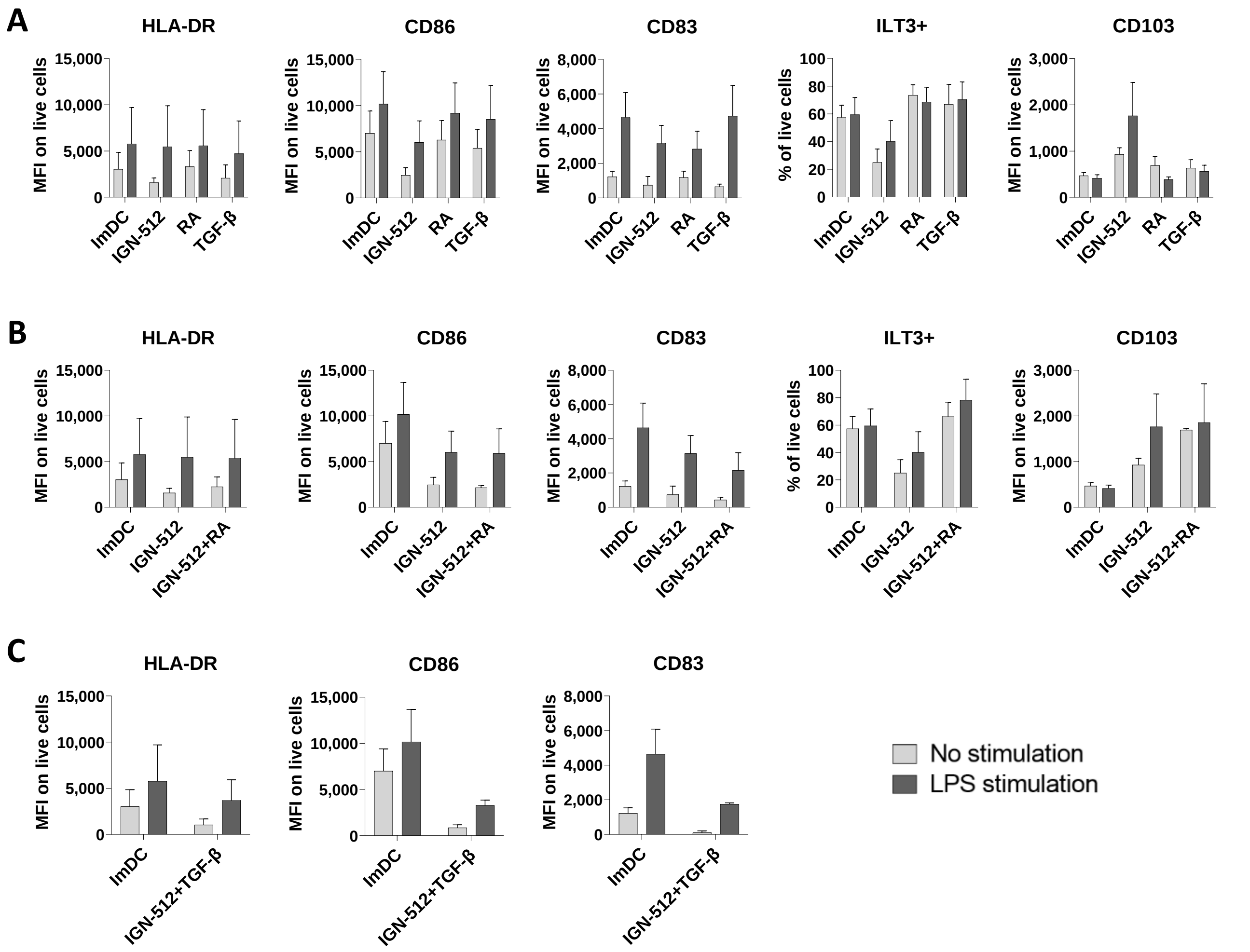

Supplementary Figure 5. Phenotypic and functional assessment of DCs differentiated in the presence of IGN-512, RA and/or TGF-β using flow cytometry. **(A)** MFI levels of HLA-DR, CD86, CD83, and CD103 on live cells and frequency of ILT3+ cells of live cells are presented for imDCs and DCs differentiated in the presence of IGN-512, RA or TGF-β without and with LPS stimulation to induce maturation. **(B)** MFI levels of HLA-DR, CD86, CD83 and CD103 on live cells and frequencies of ILT3 of live cells are presented for imDCs and DCs differentiated in the presence of IGN-512 or IGN-512+RA without and with LPS stimulation to induce maturation. **(C)** MFI levels of HLA-DR, CD86 and CD83 on live cells are presented for imDCs and DCs differentiated in the presence of IGN-512+TGF-β without and with LPS stimulation to induce maturation. Data are presented as mean with SD (A-C with n=3-4), and one-way ANOVA with post-hoc testing was used for statistical comparison

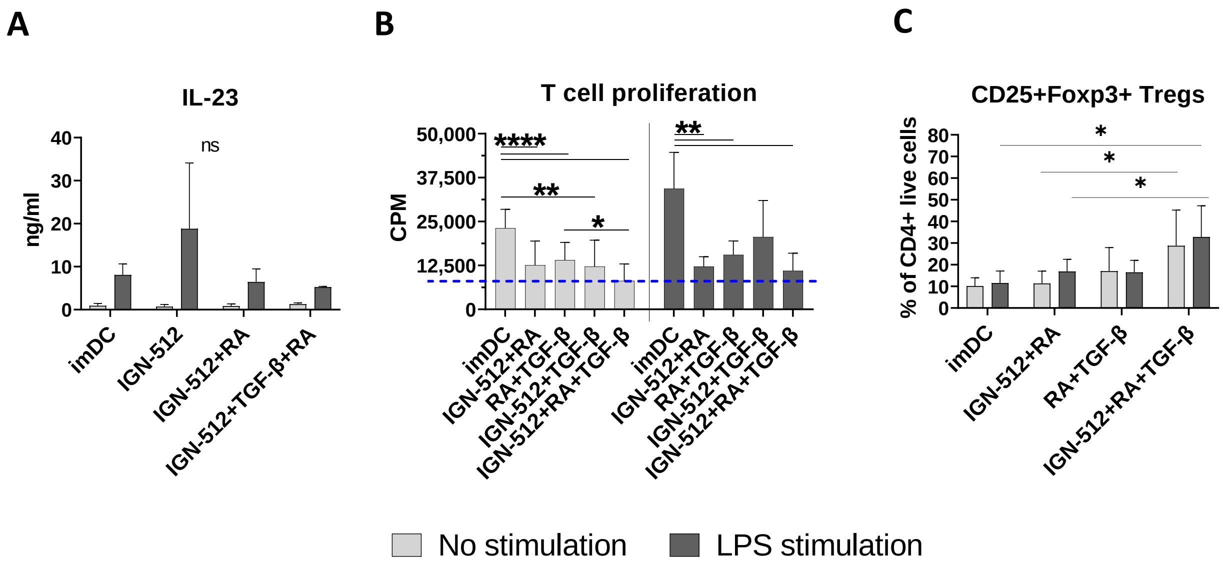

Supplementary Figure 6. Phenotypic and functional assessment of DCs differentiated in the presence of IGN-512, retinoic acid (RA) and/or TGF-β using flow cytometry. **(A)** IL-23 released in the DC culture supernatants from imDCs, and DCs differentiated in the presence of IGN-512, IGN-512+RA or IGN-512+RA+TGF-β without and with LPS stimulation to induce maturation. **(B)** T cell proliferation induced by imDCs or DCs treated with IGN-512+RA, RA+TGF-β, IGN-512+TGF-β, or IGN-512+RA+TGF-β was assessed in an MLR and was determined by incorporation of [^{3}H]-thymidine. Blue dashed line (---) represent the lowest counts per minute (CPM) which belongs to DCs differentiated in the presence of IGN-512+RA+TGF-β. **(C)** Frequencies of CD25+Foxp3+ Tregs of CD4+ T cells in an MLR with imDCs or DCs treated with IGN-512+RA, RA+TGF-β or IGN-512+RA+TGF-β. Data are presented as mean with SD (A with n=3-6, and B-C with n=6), and t-test was used for statistical comparisons. Statistical significances are presented as ns=non-significant, *=p<0.05, **=p<0.01, and ****=p<0.0001.

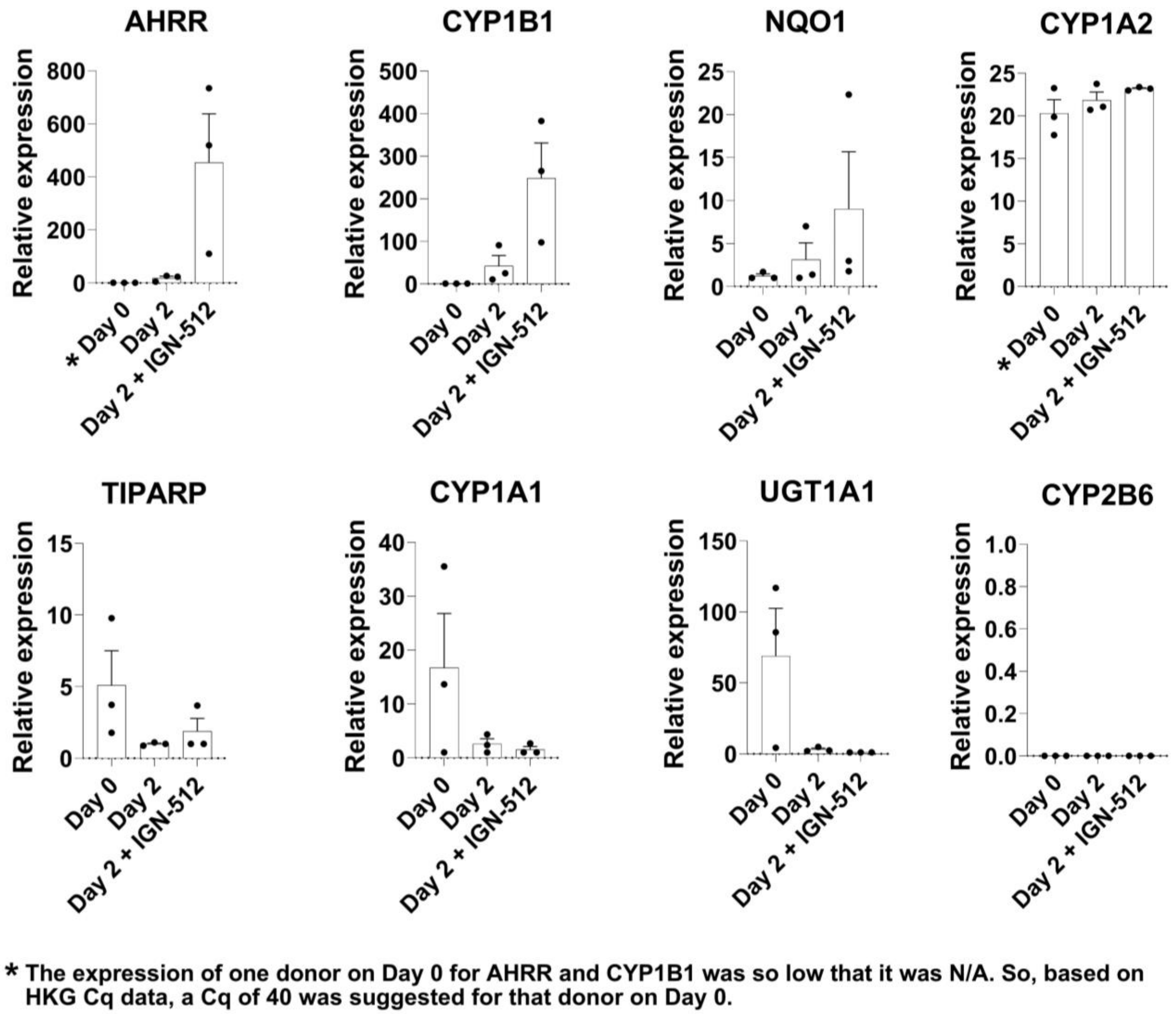

Supplementary Figure 7. The relative expression of AhR activation associated genes on non-treated monocytes, cells treated with or without the AHR agonist IGN-512. The figure shows the expression on monocytes at Day 0 and on Day 2 with and without stimulation using IGN-512. Some genes are higher expressed in IGN-512-treated cells compared to untreated cells and monocytes (AHRR, CYP1B1, and NQO1). Meanwhile, some genes are less expressed in differentiating cells on Day 2 compared to monocytes). Relative expression was calculated as $2^{-\Delta\Delta CT}$ values (normalized to gene expression of the housekeeping genes: *B2M* and *RPL13a*). Data are presented as mean with SEM where n=3.

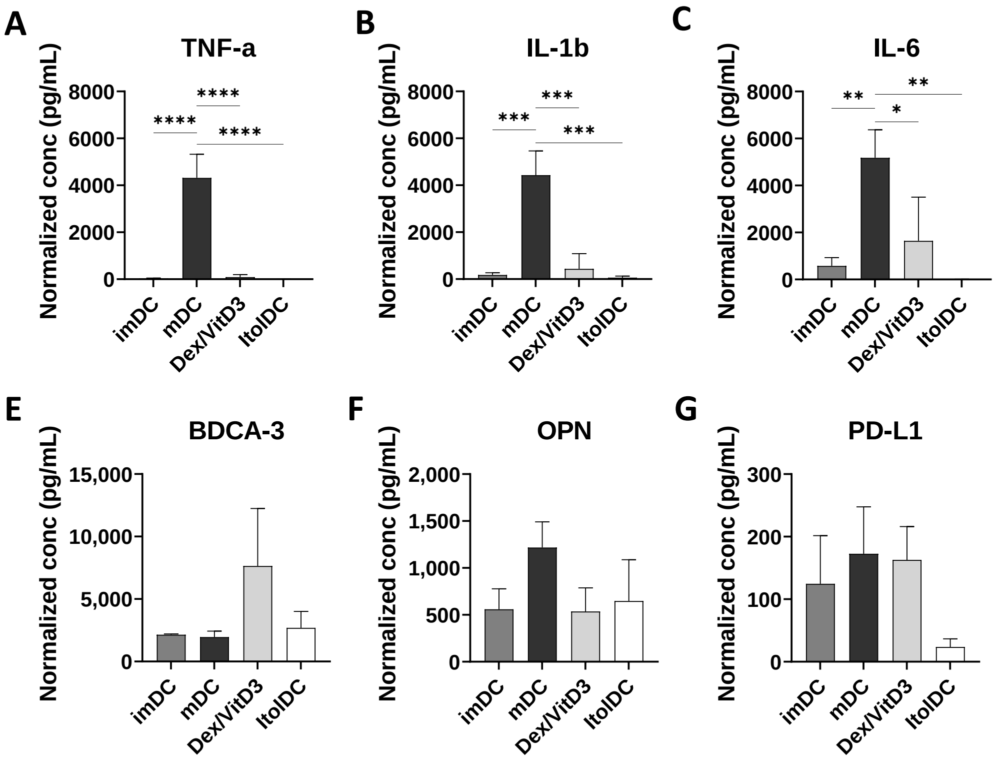

Supplementary Figure 8. Cytokine secretion from DCs. **(A-G)** Released TNF-α, IL-1β, IL-6, BDCA-3, OPN, and PD-L1 determined in the supernatants from imDC, mDC (matured with TIP6 cocktail), Dex/VitD3-tolDC and ItolDC cultures after 7 days of differentiation. Cytokines were determined using a magnetic bead-based multiplex assay, and cytokine concentrations were normalized to the number of cells in culture. Data are presented as mean with SD where n=3, and one-way ANOVA with post-hoc testing was used for statistical comparisons. Statistical significances are presented as *=p<0.05, **=p<0.01, ***=p<0.001, and ****=p<0.0001.

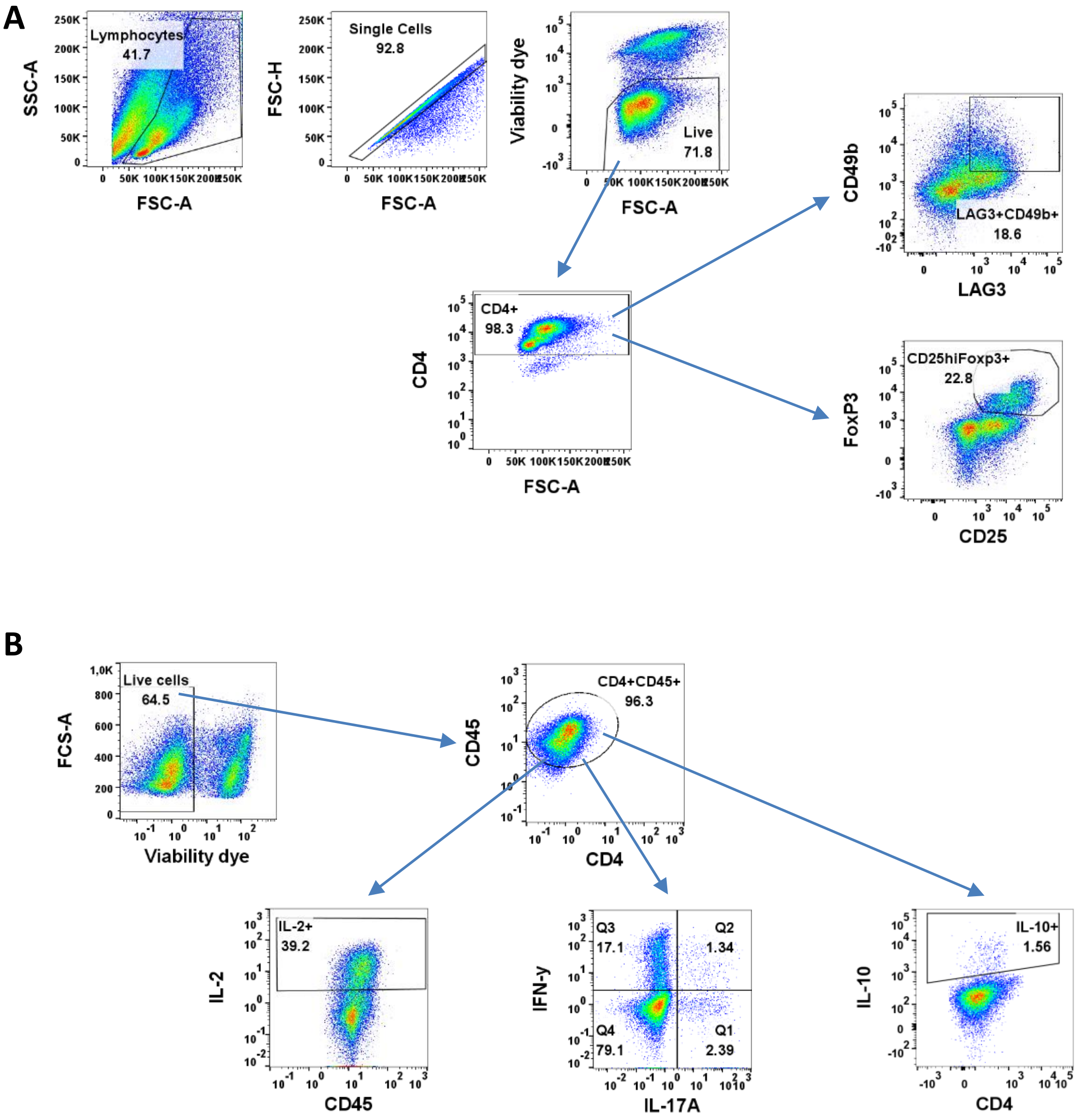

Supplementary Figure 9. Gating strategy for T cells after culture with DCs. **(A)** Tr1 and Treg. **(B)** Intracellular cytokine staining.

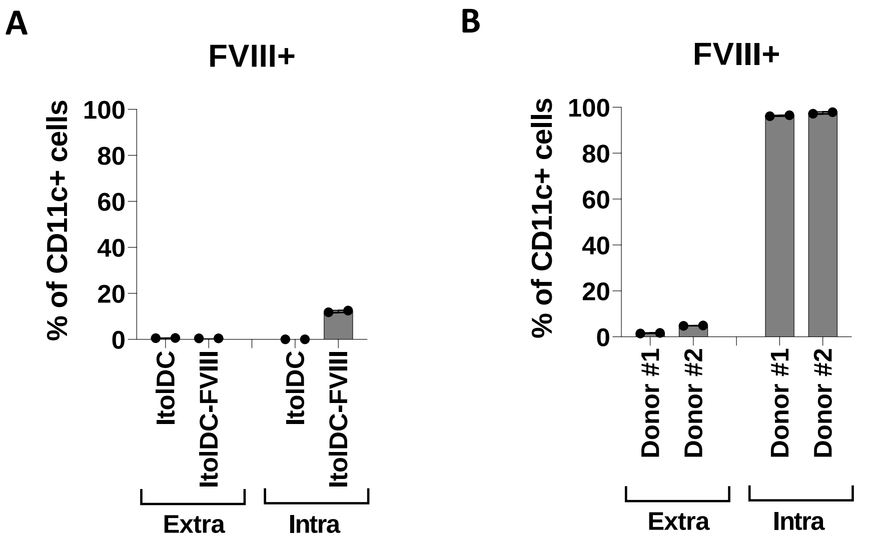

Supplementary Figure 10. FVIII-loading and detection of FVIII-uptake by ItolDCs. **(A)** Assessment of intracellular FVIII-detection assay using ItolDCs loaded with protocol for suboptimal FVIII-loading. ItolDCs without antigen-loading and of FVIII-loaded ItolDCs shows no/low frequencies of extracellular FVIII-positive cells. For the intracellular assay, FVIII-loaded ItolDCs show intracellular FVIII of ~15% while ItolDCs without antigen-loading showed no/low frequencies of intracellular FVIII. **(B)** FVIII-loaded ItolDCs of two donors have low frequencies of extracellular FVIII and high frequencies of intracellular FVIII. Data are presented as mean with SD where n=2.
